# Isolation, complete characterization and phylogeography of the first bacteriophage against Vibrio neocaledonicus, which encodes a pyruvate phosphate dikinase and represents a novel viral family

**DOI:** 10.1099/mgen.0.001403

**Published:** 2025-04-28

**Authors:** Xin Chen, Ruize Liu, Wei Wang, Yundan Liu, Jianhua Sun, Hongbing Shao, Andrew McMinn, Min Wang, Yantao Liang

**Affiliations:** 1 College of Marine Life Sciences, Institute of Evolution and Marine Biodiversity, MOE Key Laboratory of Evolution and Marine Biodiversity, Frontiers Science Center for Deep Ocean Multispheres and Earth System, Center for Ocean Carbon Neutrality, Ocean University of China, Qingdao, PR China; 2 Department of Intensive Care Unit, Qingdao Hospital, University of Health and Rehabilitation Sciences (Qingdao Municipal Hospital), Qingdao, PR China; 3 Haide College, Ocean University of China, Qingdao, PR China; 4 UMT-OUC Joint Centre for Marine Studies, Qingdao, PR China; 5 Institute for Marine and Antarctic Studies, University of Tasmania, Hobart, TAS, Australia

**Keywords:** comparative genomic analysis, phylogenetic analysis, phylogeography, pyruvate phosphate dikinase, vibriophage

## Abstract

*Vibrio* are widely distributed in aquatic environments and are major pathogens commonly found in aquaculture environments, playing a significant role in human production activities and maintaining ecological stability. Here, a novel phage, vB_VneS_J26, which infects *Vibrio neocaledonicus*, was isolated from coastal seawater in Qingdao, China. Transmission electron microscopy revealed that vB_VneS_J26 exhibits siphovirus morphotype, with a linear double-stranded DNA genome of 82,477 bp in length and G+C content of 45.11 mol%, encoding 122 putative ORFs. Three auxiliary metabolic genes related to carbon metabolism and host cell redox processes were identified, including a pyruvate phosphate dikinase, which catalyses the reversible conversion between phosphoenolpyruvate and pyruvate and is rarely detected in viruses. Whole-genome phylogenetic and comparative genomic analyses suggested that vB_VneS_J26 represents a potential novel viral family, comprising six isolated vibriophages, proposed as *Modirecodeviridae*. Phylogeographic analysis indicated that *Modirecodeviridae* is primarily distributed in epipelagic and mesopelagic zones of the Arctic and temperate tropical oceans.

­

**Impact Statement**

*Vibrio* and vibriophage serve as widely studied model systems for virus–host interactions. Although *Vibrio* is abundant in marine environments, the number of isolated vibriophages is significantly lower than that of *Vibrio*. This study describes a novel phage, vB_VneS_J26, infecting *Vibrio neocaledonicus*, revealing its phylogeny and phylogeography in marine environments. The vB_VneS_J26 is the first *V. neocaledonicus* phage containing an uncharacterized pyruvate phosphate dikinase gene, representing a novel viral family, *Modirecodeviridae*. This study provides insights into the genome, diversity, evolution, distribution and virus–host interactions of the novel *Modirecodeviridae*.

## Data Summary

The genome sequence of phage vB_VneS_J26 has been deposited in GenBank under accession number PP534163. The 16S rRNA sequences of the host C1 and J17 have also been deposited in NCBI under accession numbers PQ451821 and PQ451848 (https://www.ncbi.nlm.nih.gov/genbank/).

## Introduction

Viruses exist in nearly every ecological niche on Earth, from human gut microbiomes to deep-sea trenches, hydrothermal vents and ancient human fossils [1,2]. As key regulators of microbial communities, viruses affect microbial abundance and community structure through infecting and lysing host cells [1]. Viruses also transfer genetic material to bacteria or other microbes, increasing genetic diversity within microbial communities [3]. Viruses are integral to the microbial world and have promoted the evolution of eukaryotes, but they have also played roles in Earth’s mass extinction events [4,5]. Cultivable viruses are isolated and purified using the double-layer agar method, and their diverse morphologies can be observed through transmission electron microscopy (TEM). Advances in next-generation sequencing (NGS) have further expanded our understanding of viral genomic diversity, leading to the discovery of novel viral groups that traditional isolation techniques may have overlooked [6].

The genus *Vibrio*, a member of the family *Vibrionaceae*, comprises Gram-negative, curved rod-shaped *Gammaproteobacteria* that are widely distributed in estuarine, coastal and marine environments [7]. *Vibrio* expresses key proteins such as pili, lectins and flagella, which contribute to pathogenicity and facilitate symbiotic colonization [8], and they produce bioactive compounds with antibacterial, anticancer and antiviral properties, in addition to forming biofilms that support survival in extreme conditions [9]. *Vibrio neocaledonicus* is of particular interest due to its dual ecological and economic significance. As a virulent pathogen, *V. neocaledonicus* infects commercially vital marine organisms including the nematode (*Urechis unicinctus*), Pacific white shrimp (*Litopenaeus vannamei*) and *Yesso scallops* – the latter of which has led to significant economic losses in aquaculture [10,11]. Moreover, the extracellular polysaccharides produced by *V. neocaledonicus* exhibit properties comparable to industrial coatings, such as electroless nickel plating, underscoring its high biotechnological potential [12]. Despite its significance, no complete genome sequence of a phage infecting *V. neocaledonicus* has been deposited in GenBank, limiting our understanding of phage-bacteria interplay and the development of phage-based biocontrol strategies. Notably, the potential of phage therapy in aquaculture has been recently highlighted [13], emphasizing the need to isolate and genomically characterize these phages to better understand their therapeutic applications and reconcile the ecological impact with biotechnological exploitation.

Pyruvate phosphate dikinase (PPDK) is a rate-limiting enzyme in the Embden-Meyerhof-Parnas (EMP) glycolysis, catalysing the reversible conversion of pyruvate and phosphate to phosphoenolpyruvate (PEP), thereby regulating ATP synthesis or consumption. In C4 plants, PPDK constitutes up to 10% of total leaf protein and enhances CO_2_ fixation efficiency, making it essential to the photosynthetic pathway [14]. PPDK has also been found in various bacteria and protozoa. In prokaryotes, PPDK functions similarly to pyruvate kinase, catalysing the glycolytic reaction towards energy production [15]. Inhibitors targeting *Entamoeba* PPDK have been proposed, highlighting its potential as a therapeutic target [16]. To date, only a few phages have been reported to carry PPDK [17,18], and there have been no detailed studies on the structure and function of PPDK in viruses.

In this study, we isolated and characterized a novel vibriophage vB_VneS_J26 encoding a putative PPDK from nearshore seawater in Qingdao, China. Morphological, growth, host range, genomic and phylogenetic analyses of vB_VneS_J26 were performed. Genomic and phylogenetic analyses suggested that it is to be classified into a novel siphoviral cluster with six isolated vibriophages, representing a novel viral family, *Modirecodeviridae*. This study enhances our understanding of viral genomic features and taxonomic evolution and provides new insights into interactions between viruses and *Vibrio*.

## Methods

### Isolation of vB_VneS_J26

The phage vB_VneS_J26 was isolated in July 2023 from coastal seawater near Qingdao, China (120.42923° E 36.05505° N). The host strain used in this study, *V. neocaledonicus* C1, was provided by Professor Zhang Yuzhong’s laboratory at Shandong University and was incubated in liquid 2216E medium at 28 °C. The 16S rRNA sequence of the host strain was most similar to *V. neocaledonicus* strain NC470 (shared identity: 99.45%).

Fifty kilodaltons of cartridges in a tangential flow filtration system (Pellicon^®^ XL Cassette, Biomax^®^ 50 kDa; polyethersulfone, Millipore Corporation, Billerica, MA, USA) were used to concentrate 50 l of seawater to 200 ml. The concentrated solution was then filtered through a 0.22 µm micropore filter (Millex-GP Filter, 0.22 µm, PES 33 mm). The viral concentration was retained in 50 ml polyethylene centrifuge tubes and stored in the dark at 4 °C.

The double-layer plaque method was used in phage isolation. In a sterile environment, 200 µl of logarithmic-phase bacterial culture was mixed with 200 µl of concentrated solution and allowed to stand at 28℃ for 25 min. Then, 3.5 ml of 40 °C molten 2216E semi-solid medium (peptone 5 g l^−1^, yeast extract 1 g l^−1^, sea salt 30 g l^−1^, agar powder 7.5 g l^−1^ and pH 8.5–9) was added to the mixture. After vortex mixing, the mixture was poured onto the surface of 2216E solid medium and incubated at 28 °C for 24 h to observe the formation of phage plaques. If plaques were observed, single plaque areas were picked out and transferred to 1 ml of SM buffer [100 mM NaCl, 8 mM MgSO4·7H2O, 50 mM Tris-HCl (pH 7.5)]. The mixture was vortexed for 3 min and filtered through a 0.22 µm microporous filter. The process was repeated three times. The virus suspension was stored at 4 °C in the dark.

### Purification and concentration of vB_VneS_J26

The 1 ml virion suspensions were added to a 3 ml logarithmic-phase host culture at 28 °C for 36 h. Then, the culture was progressively scaled up to 10, 50 and 200 ml and 1 l. After completing a series of cultivations, the 1 l culture was incubated with 1 M NaCl in the dark at 4 °C for 30 min. The mixture was then centrifuged at 10,000 ***g*** for 15 min at 4 °C to remove cells and cell debris. The supernatant was collected, filtered through a 0.22 µm micropore filter and treated with 10% (wt/vol) PEG8000 at 4 °C overnight to precipitate the concentrated viral particles. The PEG8000-treated lysate was centrifuged at 10,000 ***g*** for 1 h at 4 °C, and the supernatant was carefully discarded without disturbing the pellet [19]. The pellet was resuspended in 5 ml SM buffer to obtain concentrated virion suspensions stored in the dark at 4 °C.

### Transmission electron microscopy

TEM was used to characterize the morphology of the phage [20]. A 20 µl aliquot of concentrated purified virion suspensions (~10^9^ p.f.u. ml^−1^) was spotted onto a carbon-coated copper grid and allowed to adsorb for 1 min, then air-dried. The virion suspensions were negative-stained with 2% phosphotungstic acid (pH 7.5) for 5 min and then observed under TEM (JEOL JEM-1200EX, Japan) at 100 kV and magnifications ranging from ×150,000 to ×300,000. Morphological measurements were performed using ImageJ (v1.54). A grating with known line spacing was used as an internal calibration standard to ensure accurate size measurements.

### Host range detection, one-step growth curve determination, temperature and pH stability

A cross-infectivity detection was performed using eight *Vibrio* strains to assess the host range of vB_VneS_J26. Subsequently, 200 µl of logarithmic-phase bacterial culture was mixed with 3.5 ml of 2216E semi-solid medium. The mixture was poured onto 2216E solid medium plates and allowed to cool and solidify. The concentrated purified virion suspensions were spotted on the surface of each bacterial culture plate and incubated overnight at 28 °C. The presence of phage plaques was then checked [21].

To determine the one-step growth curve, the host was mixed with the concentrated purified virion suspensions with m.o.i. of 0.01 and incubated at 28 ℃ for 20 min. To remove non-adsorbed viral particles, the mixture was centrifuged at 10,000 ***g*** at 4 °C for 1 min (repeated thrice). The pellet from the final centrifugation was resuspended in 30 ml of 2216E liquid medium. Culture was taken every 5 min for the first 30 min, every 10 min from 30 to 60 min and every 30 min from 60 to 180 min. Plaques were determined using the double-layer plaque method, with the burst size calculated as the number of plaques at different time points and quantified to reflect viral growth. Each sampling was performed in triplicate to ensure accuracy. To assess viral stability at different pH levels, 1 ml of virion suspensions (initial titer ~10^9^ p.f.u. ml^−1^) was adjusted to various pHs using HCl and NaOH. Eleven different pH solutions ranging from 2 to 12 were prepared, and the virion suspensions were incubated at 28℃ for 2 h. Two hundred microlitres of the diluted virion suspensions at different pH levels were mixed with 200 µl of the host culture. The mixture was vortexed with 3.5 ml of 2216E semi-solid medium and poured onto 2216E solid medium plates, which were incubated overnight at 28 °C. Each pH level was tested in triplicate to ensure accuracy. To evaluate viral stability at different temperatures, virion suspensions were incubated at −20 °C, 4 °C, 25 °C, 35 °C, 45 °C, 55 °C, 65 °C, 75 °C and 85 °C for 2 h. Two hundred microlitres of the diluted virion suspensions at different temperatures were mixed with 200 µl of the host culture. The mixture was vortexed with 3.5 ml of 2216E semi-solid medium and poured onto 2216E solid agar plates, which were incubated overnight at 28 °C. Each temperature was tested in triplicate to ensure accuracy. Phage plaques were counted, and the growth columns at different temperatures and pH were plotted.

### Phylogenetic analysis of the host

To obtain the 16S rRNA gene sequence, 3 ml of purified host bacterial culture in the logarithmic growth phase was centrifuged at 4 °C and 12,000 ***g*** for 5 min. The supernatant was discarded, and the pellet was sent to Sangon Biotech (Shanghai) Co., Ltd., where the universal bacterial 16S rRNA primers 27F and 1492R were used for PCR amplification and bidirectional sequencing [22]. The 16S rRNA gene sequence of the host *V. neocaledonicus* C1 was submitted to blastn (v2.14.0^+^), and a search was conducted against the core nucleotide (core_nt) database (https://ncbiinsights.ncbi.nlm.nih.gov/2024/07/18/new-blast-core-nucleotide-database/). The search identified 16S rRNA genes from *Vibrio* strains similar to C1. A total of 72 reference sequences of *Vibrio* 16S rRNA genes, including *V. neocaledonicus* J17, were downloaded. The multiple sequence alignments were performed using muscle with the Super5 algorithm [23]. After alignment, the sequences containing more than 70% gaps were removed using trimAl [24]. Duplicate sequences were filtered based on sequence content (-n) using SeqKit rmdup [25]. Phylogenetic analysis was conducted using IQ-TREE 2 (v2.2.2.7) [26], with 3,000 bootstrap replicates. The phylogenetic tree was visualized using the interactive Tree of Life (iTOL v6.9.1) (https://itol.embl.de/) [27].

### Viral genome sequencing and annotation

Viral DNA extraction for vB_VneS_J26 was performed using a viral DNA extraction kit (OMEGA Bio-tek, USA) [28]. NGS of the viral genome was conducted with the support of BioZeron Biotechnology Co., Ltd. (Shanghai, China). The concentration and quality of nucleic acids were measured using a Thermo Scientific NanoDrop spectrophotometer [29]. An Illumina TruSeq Sample V Prep Kit was used to construct paired-end (PE250) libraries with an insert size of 400 bp, and sequencing was carried out on an Illumina NovaSeq 6000 platform (150 bp×2). Raw paired-end reads were trimmed and quality-controlled using Trimmomatic (v. 0.3.6) with the following parameters: SLIDINGWINDOW:4 : 15 and MINLEN:75 [30]. High-quality clean reads were assembled using ABySS (http://www.bcgsc.ca/platform/bioinfo/software/abyss) (v1.3.7), and any gaps were filled with GapClose (v1.12) using default parameters (https://sourceforge.net/projects/soapdenovo2/files/GapCloser/) [31]. The completeness of the viral genome assembly, particularly the presence of terminal repeats, was assessed using CheckV (v1.7.0) [32]. PhageTerm v1.0.11 was used to identify termini, with the reads showing the maximum coverage being considered as viral termini [33]. Putative ORFs were predicted using RAST (https://rast.nmpdr.org/rast.cgi) [34] and PRODIGAL (v2.6.3) [35]. ORFs were annotated through blastp (v2.14.0+) against the nr database (https://blast.ncbi.nlm.nih.gov/), as well as with hidden Markov model-based searches against the Pfam-A (v35.0) [36] and KEGG database (http://www.genome.jp/kegg/) [37]. Additionally, HHpred (https://toolkit.tuebingen.mpg.de/tools/hhpred) was used to detect conserved protein domains in each ORF [38]. The results from HHPred were manually curated, with an E-value threshold of 1e-5 and sequence identity >30%. Annotation information from different databases was manually cross-checked. tRNAs were identified using tRNAscan-SE (https://lowelab.ucsc.edu/tRNAscan-SE) [39]. The genome map was performed in CLC Genomics Workbench 20.0 in master view mode. G-C skew was calculated and visualized using SkewIT (v1.0) [40].

### Tetranucleotide correlation analysis

A total of 27 fragments were extracted from the vB_VneS_J26 genome (window size: 10 kbp; step size: 3 kbp). To maintain genome integrity, the fragments were first concatenated, ensuring that each 3 kbp section was included in the corresponding 10 kbp fragment [41]. For each fragment, the frequencies of all 256 possible tetranucleotide combinations (ranging from ‘AAAA’ to ‘TTTT’) were calculated and normalized using the *Z*-score algorithm. Pearson correlation coefficients (R values) were computed based on a comparison of the *Z*-scores across each fragment and the entire viral genome [42].

### Protein three-dimensional structure prediction and comparison

The three-dimensional structure of ORF98 was predicted using the AlphaFold server of Google DeepMind (https://alphafoldserver.com/) [43], with the parameters set to automatic and the best model selected based on the highest ipTM score. The predicted Protein Data Bank (PDB) file was obtained and visualized using PyMOL 3.0. Structure-based searches were performed using the Dali server (http://ekhidna.biocenter.helsinki.fi/dali/) [44]. The molecular structure of PEP was acquired from PubChem (https://pubchem.ncbi.nlm.nih.gov/). Ligand docking was visualized using Discovery Studio 2019. The three-dimensional structure of PPDK (PDB ID: 1GGO) was used as a template, and structure alignment with ORF98 was performed using the PyMOL alignment plugin [45]. Following the structure alignment, structural optimization was carried out using the least square method, iterating to select the aligned structural model with the lowest Root Mean Square Deviation (RMSD) value [46].

### Viral genomic diversity expansion of vB_VneS_J26

A total of 18,671 reference viral genomes were downloaded from GenBank. Redundant viral genomes were removed by clustering using MMseqs2 (minimum identity: 99%; coverage mode: 5; coverage identity: 100%; cluster mode: 2) [47]. An all-to-all blastp (e-value<1e-5) comparison was performed on all proteins, driven by Diamond. Proteins from reference viruses closely related to the vB_VneS_J26 genome were clustered using the Markov clustering algorithm (MCL), generating protein clusters (PCs) based on the E-values from the all-to-all blastp. The relationship weights between different genomes were described using similarity scores calculated by vConTACT (v2.0) [48]. Viral clusters (VCs) were assigned using ClusterONE [49]. All genomes with weighted connections to vB_VneS_J26 were selected for network analysis and visualized using Gephi (v0.9.7). A proteome tree was constructed using ViPTree (https://www.genome.jp/viptree/) [50], and a comparative genomic analysis of *Modirecodeviridae* was performed through tblastx. Comparative genomic analysis was visualized using DiGAlign (v1.3). A whole-genome phylogenetic tree based on nucleotide sequences was constructed using the Virus Classification and Tree Building Online Resource (VICTOR) (https://victor.dsmz.de) [51], applying the GBDP_D6 model, and visualized using iTOL v6. The average nucleotide identity (ANI) of *Modirecodeviridae* was calculated using the Virus Genome Distance Calculator (VIRIDIC) [52], showing nucleotide-level homology and supporting genus and species delineation. A maximum-likelihood phylogenetic tree was generated using IQ-TREE 2 (v2.2.2.7) and visualized with iTOL v6. Homologous viruses were defined as those forming a monophyletic group on the phylogenetic tree, and viruses in monophyletic clades were used for subsequent analyses.

### Phylogeography of *Modirecodeviridae* in global ocean

The metagenomic tool minimap2 was employed to analyse the relative abundance of *Modirecodeviridae* based on TPM (Transcripts Per Kilobase of exon model per Million mapped reads) values [53]. The Global Ocean Virome 2 (GOV2) dataset provided insights into the biogeographical distribution of *Modirecodeviridae* in marine environments [54]. This dataset is categorized into five viral ecological zones (VEZs) – Antarctic, Arctic (ARC), temperate and tropical epipelagic (EPI), bathypelagic (BATHY) and tropical mesopelagic (MES) – and includes 154 viral metagenomes [55]. Depth ranges for the EPI, MES and BATHY zones are defined as 0–150 m, 150–1,000 m and greater than 2,000 m, respectively [56]. The study compared *Modirecodeviridae* to six representative phages: *Prochlorococcus* phage P-RSM4, *Prochlorococcus* phage Syn33, *Synechococcus* phage S-RSM4, *Pelagibacter* phage HTVC019P, *Tiamatvirus* PSSP7 and Cyanophage S-TIM4, all of which are widely distributed in marine environments [57,58]. The relative abundance of *Modirecodeviridae* was calculated using CoverM (v0.3.0), with parameters set to 95% minimum reads identity and 75% minimum reads coverage. The relative abundance of these viruses was transformed using log_10_^(TPM+1)^ and visualized using bubble charts and stacked bar plots.

## Results and discussion

### Phylogeny of *V. neocaledonicus* C1 and J17

A phylogenetic tree was constructed based on the 16S rRNA gene sequences of C1, J17 and 72 other *Vibrio* reference strains (Fig. S1, available in the online Supplementary Material). The results showed that C1, J17 and *V. neocaledonicus* strain NC470 formed a monophyletic clade, with C1 being more closely related to *V. neocaledonicus* strain NC470. However, the evolutionary distance between C1 and their last common ancestor (LCA) was 0.0142, which is longer than the evolutionary distance of 0.00498 between *V. neocaledonicus* strain NC470 and the LCA. In contrast, J17 had a similar evolutionary distance to *V. neocaledonicus* strain NC470 (0.005). These findings suggest that J17 and C1 may represent two new variants of *V. neocaledonicus*.

### Morphology and characterization of vB_VneS_J26

**Fig. 1.**
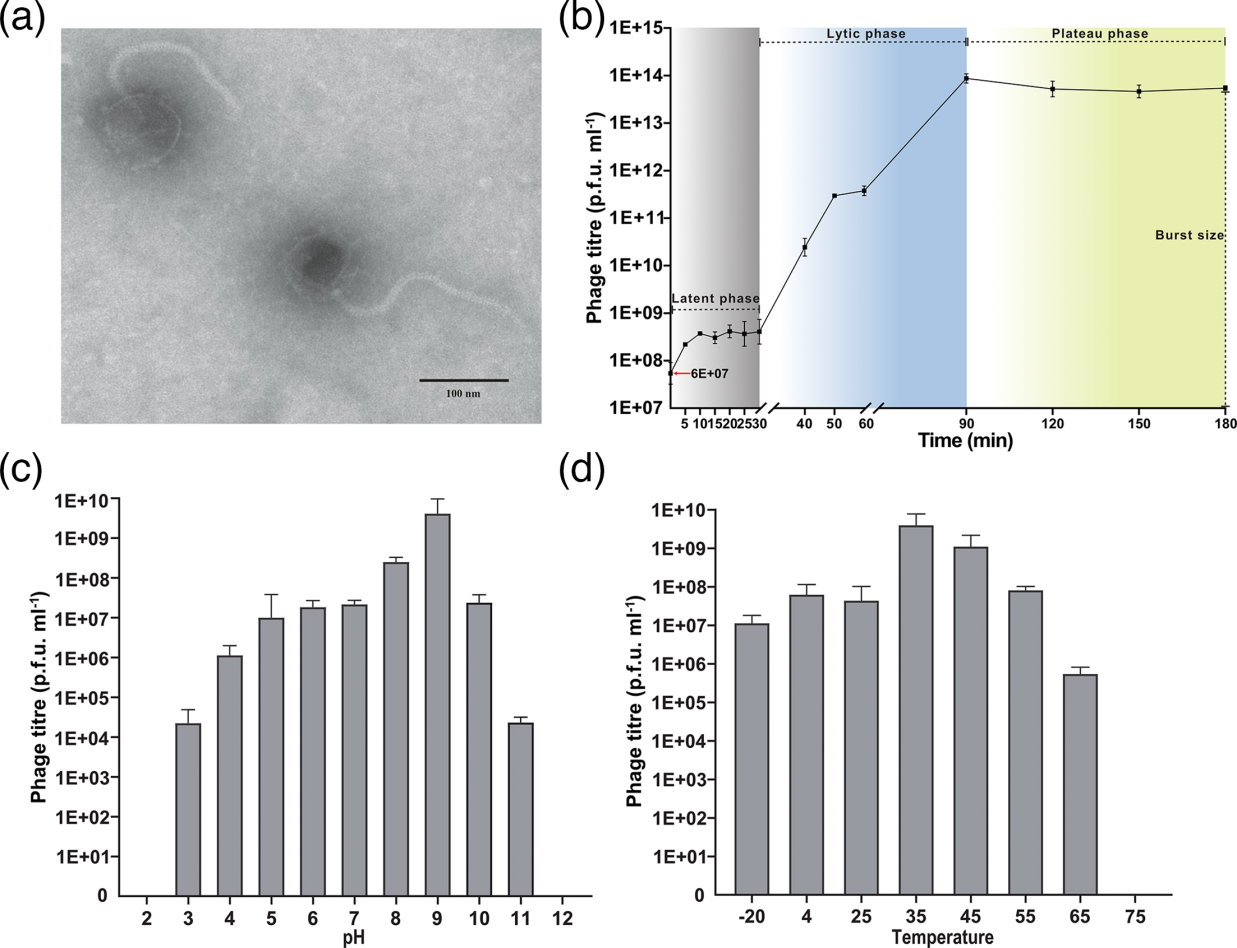
Properties of vB_VneS_J26. (**a**) Morphology of vB_VneS_J26 using TEM. (**b**) One-step growth curve of vB_VneS_J26. (**c**) pH stability of vB_VneS_J26. (**d**) Temperature stability of vB_VneS_J26.

TEM image showed that vB_VneS_J26 exhibits siphovirus morphotype, characterized by an icosahedral capsid with an average diameter of 74 nm and a long, non-contractile tail measuring ~221 nm in length (Fig. 1a). After mixing the vB_VneS_J26 with its host bacteria at an m.o.i. of 0.01, this resulted in an initial phage titre of 6×10^7^ p.f.u. ml^−1^. Following a 20 min adsorption period, the latent phase commenced. Over the next 30 min, the phage titre remained stable at ~10⁸ p.f.u. ml^−1^, during which the phage titre slowly increased, indicating the replication of viruses and synthesis of progeny. The lytic phase was detected between 40 and 90 min, where progeny viruses were released through bacterial lysis. After 90 min, the titre of vB_VneS_J26 reached its peak and plateaued. The burst size was ~105 viral particles per cell (Fig. 1b). vB_VneS_J26 exhibited optimal activity at pH 9, with activity decreasing at both higher and lower pH levels. Complete inactivation was observed at pH 2 and 12 (Fig. 1c), suggesting that vB_VneS_J26 is sensitive to highly alkaline conditions. The optimal growth temperature for vB_VneS_J26 was 35 °C. vB_VneS_J26 maintained a relatively stable titre between −20 and 25 °C, but its titre sharply declined above 35 °C, with complete inactivation observed at 75 °C (Fig. 1d).

Cross-infection tests on *Vibrio* strains revealed that out of the eight tested *Vibrio* strains, vB_VneS_J26 could infect *V. neocaledonicus* J17 and C1 (Table 1), indicating that it has a host range represented only from *V. neocaledonicus* to the best of our knowledge.

**Table 1.** Host range of vibriophage vB_VneS_J26

*Vibrio* strain	Susceptibility
*V. neocaledonicus* C1	+
*Vibrio alginolyticus*	−
*V. neocaledonicus* J17	+
*Vibrio campbellii*	−
*Vibrio jasicida*	−
*Vibrio hyugaensis*	−
*Vibrio parahaemolyticus*	−
*Vibrio stylophorae*	−

### Genomic characteristics of vB_VneS_J26

**Fig. 2.**
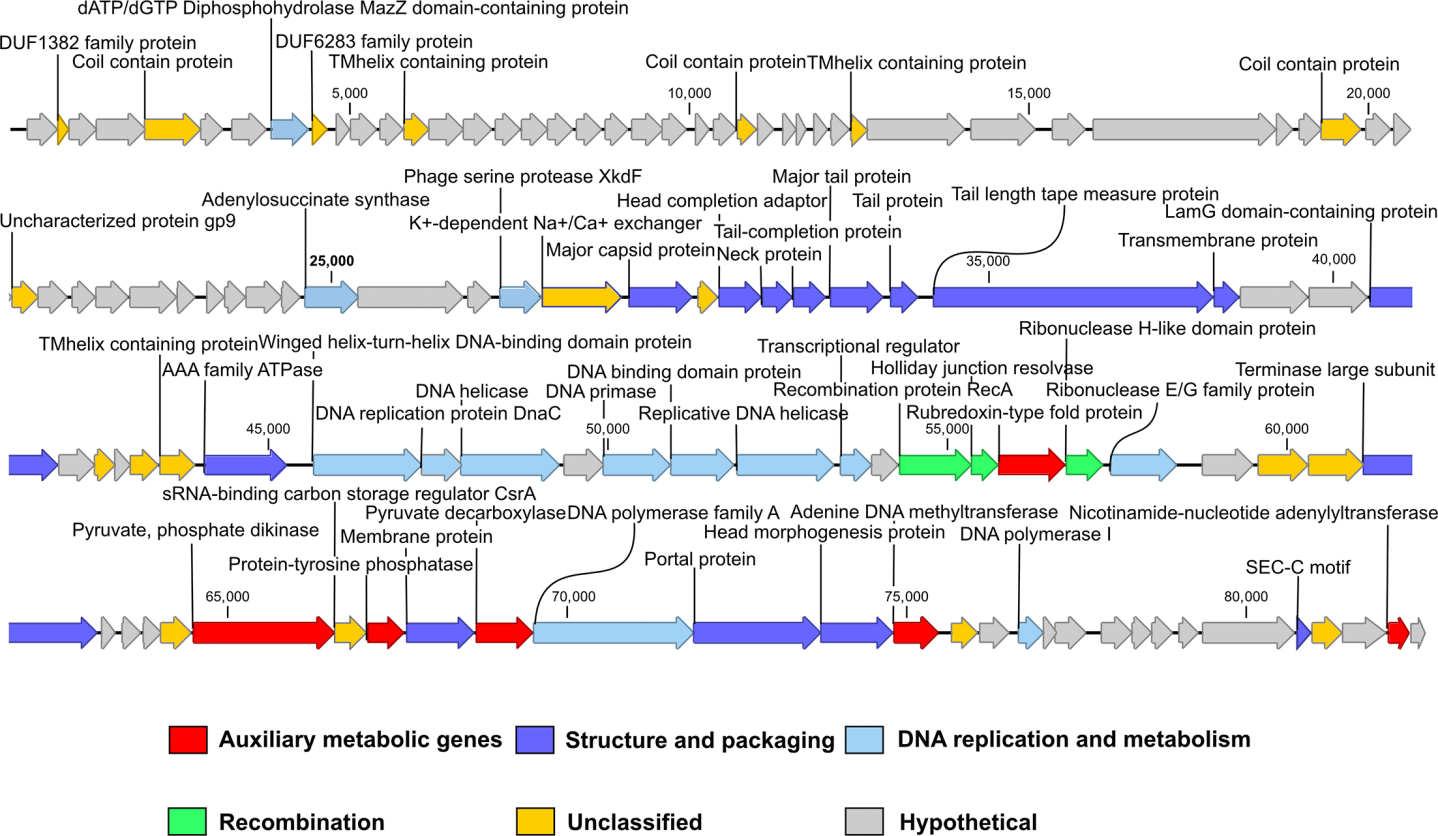
Genomic map of vB_VneS_J26. The putative functional categories are defined based on annotation and are colour-coded accordingly. The length of each arrow corresponds to the length of the respective gene.

vB_VneS_J26 possessed a linear double-stranded DNA genome with a length of 82,477 bp and a G+C content of 45.11 mol%, slightly higher than that of its host (44.55%). No tRNAs, transposases or integrases were predicted in the genome. Systematic analysis showed that the genome of J26 lacks known lysogen-related elements, which is consistent with the genomic characteristics of virulent phages. The genome is composed of 122 putative ORFs, with a coding density of 93.82%. Most ORFs start with the ATG codon, whilst five begin with GTG. Amongst the 122 ORFs, 57 match functional homologues and can be grouped into six distinct modules: structure and packaging (*n*=16), DNA replication and metabolism (*n*=16), recombination (*n*=3), virulence factors (*n*=1), auxiliary metabolic genes (AMGs, *n*=3) and 18 with known functions that could not be classified. All 122 coding genes are located on the sense strand, with no genes encoded on the antisense strand (Table S1 and Fig. 2). PhageTerm analysis identified the termini of vB_VneS_J26, revealing that its ends resemble those of P1-like phages with a headful packaging mechanism. Phages utilizing headful packaging typically produce concatemeric genomes, containing multiple copies of genomes [59]. Cumulative GC skew analysis indicates the origin and terminus of replication in the viral genome (Fig. S2). The likely origin of replication was found near the 10,400 nt, and the terminus near the 82,100 nt, where two breakpoints were detected, reflecting asymmetric base composition. Pearson correlation coefficients for each 10 kbp segment of the genome were all above 0.9 (Fig. S3), indicating a strong correlation with the overall genome and no evidence of foreign genetic substance insertions.

Amongst the 13 ORFs associated with DNA replication and metabolism (Fig. 2), ORF76–77, 79–82, 88, 103 and 109 encoded replication proteins commonly associated with viral replication and metabolism. ORF8 contained the MazZ domain of deoxyadenosine triphosphate (dATP)/deoxyguanosine triphosphate (dGTP) diphosphohydrolase, catalysing the hydrolysis of dATP or dGTP into pyrophosphate and deoxyadenosine monophosphate or deoxyguansine monophosphate. ORF52 encoded adenylosuccinate synthase (*PurA*), an enzyme crucial in purine biosynthesis, which catalyses the guanosine triphosphate (GTP)-dependent conversion of inosine monophosphate (IMP) and aspartate into phosphate and N(6)-(1,2-dicarboxyethyl)-adenosine monophosphate (AMP). In the purine nucleotide *de novo* synthesis pathway, IMP is converted into AMP using GTP, and GMP synthesis requires ATP, ensuring balanced purine nucleotide production through cross-regulation [60]. ORF55 encoded phage serine protease XkdF, whose domain is commonly found in phages, although its exact function remains unknown. However, it is speculated to be part of a serine protease superfamily involved in viral procapsid protein processing [61].

Fifteen ORFs were associated with the structural and packaging module (Fig. 2). ORF68 encoded LamG domain-containing protein, typically a Ca^2+^-mediated receptor that may be involved in signal transduction and cell adhesion. ORF118 encoded SEC-C motif protein, which is speculated to be a potential nucleic acid-binding domain.

The genome of vB_VneS_J26 contained three genes related to the recombination module (Fig. 2). ORF84 encoded recombination protein RecA, which initiates homologous recombination and DNA repair upon damage by binding to single-stranded DNA to form nucleoprotein filaments. ORF85 encoded Holliday junction resolvase (HJR), which processes Holliday junctions (HJs) – four-way DNA structures formed during homologous recombination that link damaged and undamaged DNA segments for the repair of double-strand breaks. After recombination is complete, HJR removes HJs to finalize the repair process. ORF87 encoded ribonuclease H-like domain protein, which may assist in DNA replication and repair, furthering recombination. After infecting host cells, phages may suffer genomic DNA damage from nucleases or environmental factors [62]. These enzymes associated with recombination not only enable effective DNA repair and maintain genomic integrity but also boost viral genetic diversity and adaptability [63].

ORF99 encoded sRNA-binding carbon storage regulator A (CsrA). CsrA binds to GGA motifs in target mRNAs, near ribosome binding sites in the 5′ UTR, blocking translation by preventing ribosome attachment. Campa *et al*. found that CsrA homologues in phages and mobile elements inhibit bacterial CRISPR-Cas gene expression by binding to *cas* gene mRNA, affecting translation and stability, thus suppressing bacterial defences and enhancing viral replication [64]. CsrA is a key regulatory protein in many Gram-negative bacteria, coordinating central carbon metabolism, biofilm formation, quorum sensing and virulence factor production [65].

### Six AMGs

Six AMGs are predicted in the genome of the vB_VneS_J26 (Fig. 2). ORF100 encoded protein tyrosine phosphatase (PTP), which plays key roles in cellular proliferation, differentiation and metabolism by dephosphorylating tyrosine kinases. Ibrahim *et al*. [66] suggest that viral PTPs can disrupt host cellular signalling pathways, manipulating host physiology to support rapid viral replication. PTP is crucial during the early stages of viral infection as it can reprogram host metabolic pathways, supplying the viruses with essential energy and biosynthetic precursors. ORF106 encoded adenine DNA methyltransferase, transferring methyl groups from S-adenosyl-l-methionine to adenine residues, producing methylated DNA. Phage-encoded adenine DNA methyltransferase may play roles in regulating gene expression during the viral lifecycle and protecting the viral genome from host restriction-modification systems [67,68]. ORF121 encoded nicotinamide nucleotide adenylyltransferase (NMNAT), which catalyses the reversible transfer of the adenosine portion of ATP to nicotinamide mononucleotide to synthesize NAD^+^, playing a key role in NAD *de novo* and salvage pathways. NMNAT elevation supports the host’s tricarboxylic acid (TCA) cycle and ATP production, providing the energy necessary for viral replication [69,70]. Additionally, the phage-encoded Sir2/cobB deacetylase utilizes NAD^+^ to activate the host’s acetyl-CoA synthetase, further stimulating energy metabolism [71]. By manipulating the host’s NAD^+^ metabolic pathway, the virus establishes a molecular foundation for large-scale viral genome replication.

ORF86 encoded rubredoxin-type fold protein, a simple iron-sulphur protein without inorganic sulphides, containing an [Fe(SCys)_4_] centre with iron coordinated by four conserved cysteine residues. Rubredoxin acts as a cofactor or electron carrier to help organisms resist oxidative stress and can serve as a redox enzyme to protect the host from reactive oxygen species, thereby promoting viral replication [72]. ORF98 and ORF102 encoded putative PPDK and pyruvate decarboxylase (PDC). PPDK catalyses the reversible conversion between PEP and pyruvate, a key step in the EMP pathway of glycolysis. Viruses carrying PPDK could enhance carbohydrate metabolism in the host, generating energy to produce more progeny viruses [17,73]. PDC irreversibly converts pyruvate into acetaldehyde and CO_2_, with acetaldehyde further converted into ethanol under anaerobic conditions or into acetyl-CoA under aerobic conditions to support energy production, lipid synthesis and amino acid biosynthesis. PDC, composed of two homodimer subunits with thiamine pyrophosphate and magnesium ions as cofactors, is widely present in plants and fungi, less so in prokaryotes, and has not been reported in viruses [74]. During viral infection, expression of the PDC could benefit the virus by providing energy for the release of progeny viral particles through subsequent fermentation reactions and reducing competition with the host for the common ATP pool [75,76].

### Structural and evolutionary analysis of ORF98

**Fig. 3.**
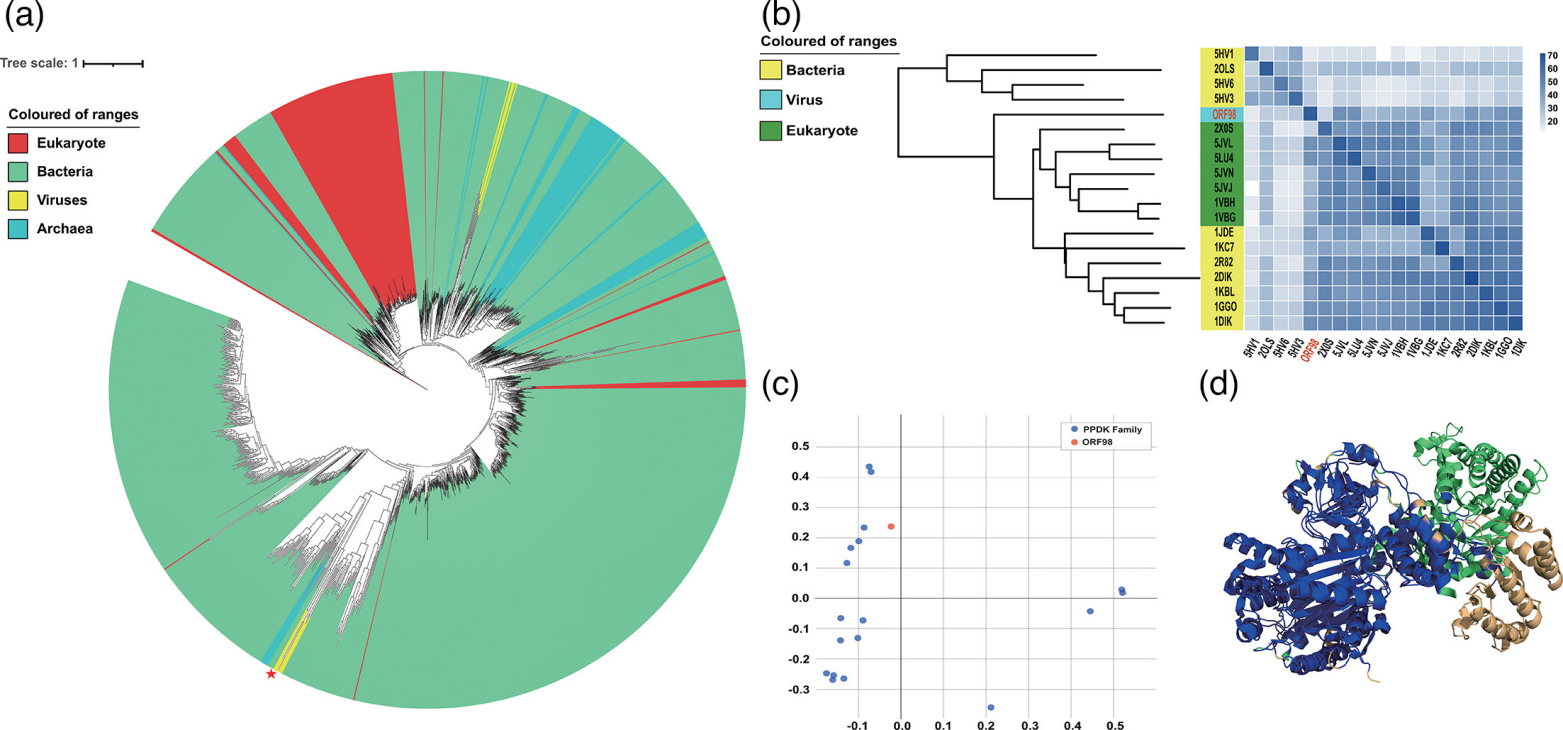
Phylogenetic and structural analyses of ORF98 and associated proteins. (**a**) A circular phylogenetic tree is constructed using the maximum-likelihood method with ORF98 and 1,431 PPDK family sequences. Green indicates bacteria; red indicates eukaryotes; yellow indicates viruses; blue indicates archaea. The red star indicates ORF98. (**b**) The matrix and clustering dendrogram are based on pairwise *Z*-scores of ORF98 and 18 PPDK families. In the dendrogram, different protein phylum is highlighted with different colours. The colour bar represents the corresponding *Z*-scores. (**c**) Correspondence analysis of PPDK family domain analysis. Data points corresponding to ORF98 are positioned relative to each other based on the similarity of structural neighbourhoods. ORF98 is represented in red. (**d**) Domain comparison of ORF98 is shown in green, and 1GGO is shown in brown. Conserved overlapping regions are shown in blue.

We retrieved proteins encoding PPDK from UniRef90 (https://www.uniprot.org/uniref), selecting 26,811 sequences from the dataset and manually removing unrelated sequences. Redundant sequences were removed using MMseqs2 (minimum identity: 99%; coverage mode: 5; coverage identity: 100%; cluster mode: 2). We constructed a circular maximum-likelihood phylogenetic tree representing 1,431 sequences and vB_VneS_J26 using IQ-TREE 2 by muscle for alignment and trimAl of PPDK family sequences. PPDKs were mainly detected in bacteria, less in eukaryotes and archaea and rarely in viruses. ORF98 clustered into a clade with six PPDK family proteins annotated in viruses (Fig. 3a). PPDK is widely detected in the phylum *Proteobacteria* according to UniProtKB, with only 30 of the 87,074 PPDK sequences found in unreviewed entry being viral in origin – two from environmental sample-assembled viral genomes, and 27 from the realm *Duplodnaviria*, with one from the phylum *Nucleocytoviricota*. Amongst the 27 isolated viruses, 20, 3, 2, 1 and 1 infect *Vibrio*, *Stenotrophomonas*, *Rhizobium*, *Rhodobacter* and *Peptoclostridium*, respectively (Table S2). The three-dimensional structure of ORF98 was predicted using the AlphaFold server from Google DeepMind, and the model with the highest ipTM score of 0.71 was selected for subsequent analysis. Protein three-dimensional structure comparison of ORF98 with the PDB coordinates was performed using the DALI server (Fig. 3b). Structural similarity between cellular and viral proteins was evaluated based on the Dali *Z*-score, which measures the quality of three-dimensional structure alignment. *Z*-score >2, indicating two sd above the expected value, is considered significant. The 18 validated PPDKs were compared in a similarity matrix and hierarchical clustering based on the average Dali *Z*-score. ORF98 shares the closest structural similarity with 1GGO (*Z*=45.7), 5LU4 (*Z*=44.2) and 5JVL (*Z*=44.1), all of which are X-ray diffraction-derived structures of PPDK. Specifically, 1GGO encodes PPDK from *Clostridium symbiosum*, whilst 5LU4 and 5JVL encode PPDK from *Flaveria trinervia*. Using the DALI server for domain analysis (Fig. 3c), ORF98 was compared to known PPDK families, and the results indicated that ORF98 is closely related to PPDK. A comparison of ORF98 and 1GGO showed that ORF98 and 1GGO are highly homologous, with conserved overlapping regions shown in blue (Fig. 3d). ORF98 and 1GGO are completely overlapping in residues 1–527. Of 691 amino acid positions, 489 were aligned with 1GGO, indicating a 70.76% similarity between ORF98 and the validated PPDK. A total of 75.2% of the predicted three-dimensional structure model for vB_VneS_J26 has a per-atom Local Distance Difference Test greater than 70 (Fig. S4). A comparison of ORF98 with the 18 PPDKs was visualized using ESPript 3 (Fig. S5). Yellow regions indicate positions with more than 70% similarity to ORF98, whilst red regions show identical positions. Secondary structure assignments were predicted by Define Secondary Structure of Proteins for ORF98 (Fig. S6). Based on the above results, ORF98 is highly homologous to the PPDK family. In total, structural and phylogenetic tree analyses indicate that vB_VneS_J26 is a novel isolated *V. neocaledonicus* phage-carrying putative PPDK.

### Three-dimensional structure and ligand binding of ORF98

**Fig. 4.**
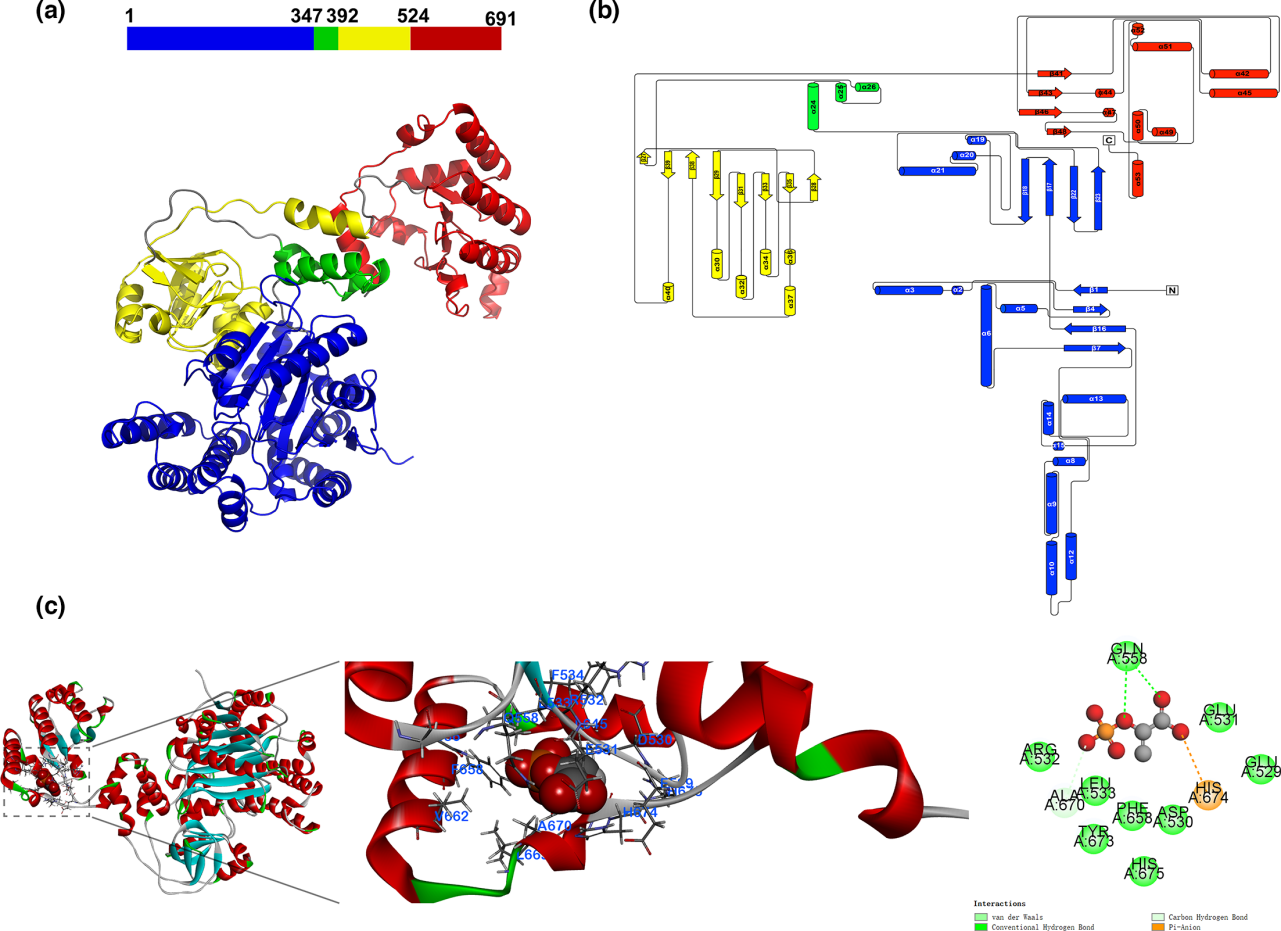
Structural and domain architecture of ORF98 and its interaction with PEP. (**a**) Domain organization and three-dimensional structure of ORF98. The NBD, LD, CD and PBD domains are coloured in blue, green, yellow and red, respectively. (**b**) Topological representation of the secondary structure of ORF98. (**c**) Three-dimensional structure of ORF98 in complex with PEP. PEP atoms are depicted as spheres, whilst the amino acids within the binding pocket are shown as sticks. The interaction between PEP and the binding pocket residues is represented in a 2D diagram.

The modular structure of PPDK and the three-dimensional structure of ORF98 contain four distinct domains (Fig. 4a): the nucleotide ATP/AMP binding domain (NBD) at the N-terminal region, encompassing residues 1–347; the PEP/pyruvate binding domain (PBD) at the C-terminal region, including residues 524–691; a linker domain (LD) between residues 347 and 392; and a central domain (CD) between residues 392 and 524, which catalyses the transfer of a phosphoryl group from ATP to pyruvate or from PEP to AMP [77]. The crystallographic structures of PPDK in different conformational states, such as in C4 plants like *Zea* and *C. symbiosum*, show that during the catalytic cycle, the catalytic histidine (H456) in the CD undergoes rotational motion to shuttle the phosphoryl group between the two active sites in NBD and PBD [78]. The NBD primarily consists of 15 *α*-helices and eight *β*-strands. The PBD contains nine *α*-helices and four *β*-strands, forming a conserved triose-phosphate isomerase (TIM) barrel topology, with four parallel *β*-strands arranged into a *β*-barrel, surrounded by nine *α*-helices, consistent with the PPDK crystal structure observed by Herzberg *et al*. [79]. The LD is composed of three *α*-helices (α24, α25 and α26). The CD, which consists of five *α*-helices and eight *β*-strands, forms a ‘swivel’ *α*/*β* sandwich structure, where the *α*-helices and *β*-strands align in parallel. The CD of PPDK from both protozoa and proteobacteria inclines towards the N-terminal nucleotide-binding domain [15]. Structural analysis of ORF98 indicates that its CD is similarly inclined towards the NBD. The third *α*-helix (α34) in the CD contains the catalytic residue H456, which plays a critical role in the phosphoryl group transfer between NBD and PBD, a residue highly conserved across PPDK from all organisms (Fig. 4b) [77]. To test the binding of PEP to ORF98, molecular docking was performed with ORF98 as the receptor protein and PEP as the molecular ligand. Potential receptor active sites were identified in the protein cavity of ORF98, particularly within the PBD region, where predicted shape-based descriptors were generated. Using the LibDock protocol in Discovery Studio, polar and non-polar regions of the predicted active sites were calculated, with a Pose Cluster Radius set to 0.5 and an RMSD threshold of 0.5 Å. The ligand was rigidly docked into each active site, and the resulting interactions were scored. The highest-scoring docking conformations were retained, filtered by the predicted Libdockscore and subsequently energy-optimized. The best-scoring docking conformation was selected as the final result and visualized. As shown in Fig. 4(c), the PEP ligand binds within the central channel of the TIM barrel in the PBD, exhibiting a binding mode similar to that proposed by Herzberg *et al*. [79]. The binding pocket of PEP consists of acidic amino acids (GLU-529, ASP-530 and GLU-531), neutral amino acids (GLN-558 and TYR-673), basic amino acids (ARG-532, HIS-674 and HIS-675) and hydrophobic amino acids (LEU-533, PHE-658 and ALA-670). These findings suggest that PEP acts as an endogenous ligand of PPDK, which, under pyruvate-limiting conditions, serves as a competitive inhibitor [80], thereby facilitating the reversible conversion between PEP and pyruvate.

### Vibriophage vB_VneS_J26 represents a novel viral family, *Modirecodeviridae*

**Fig. 5.**
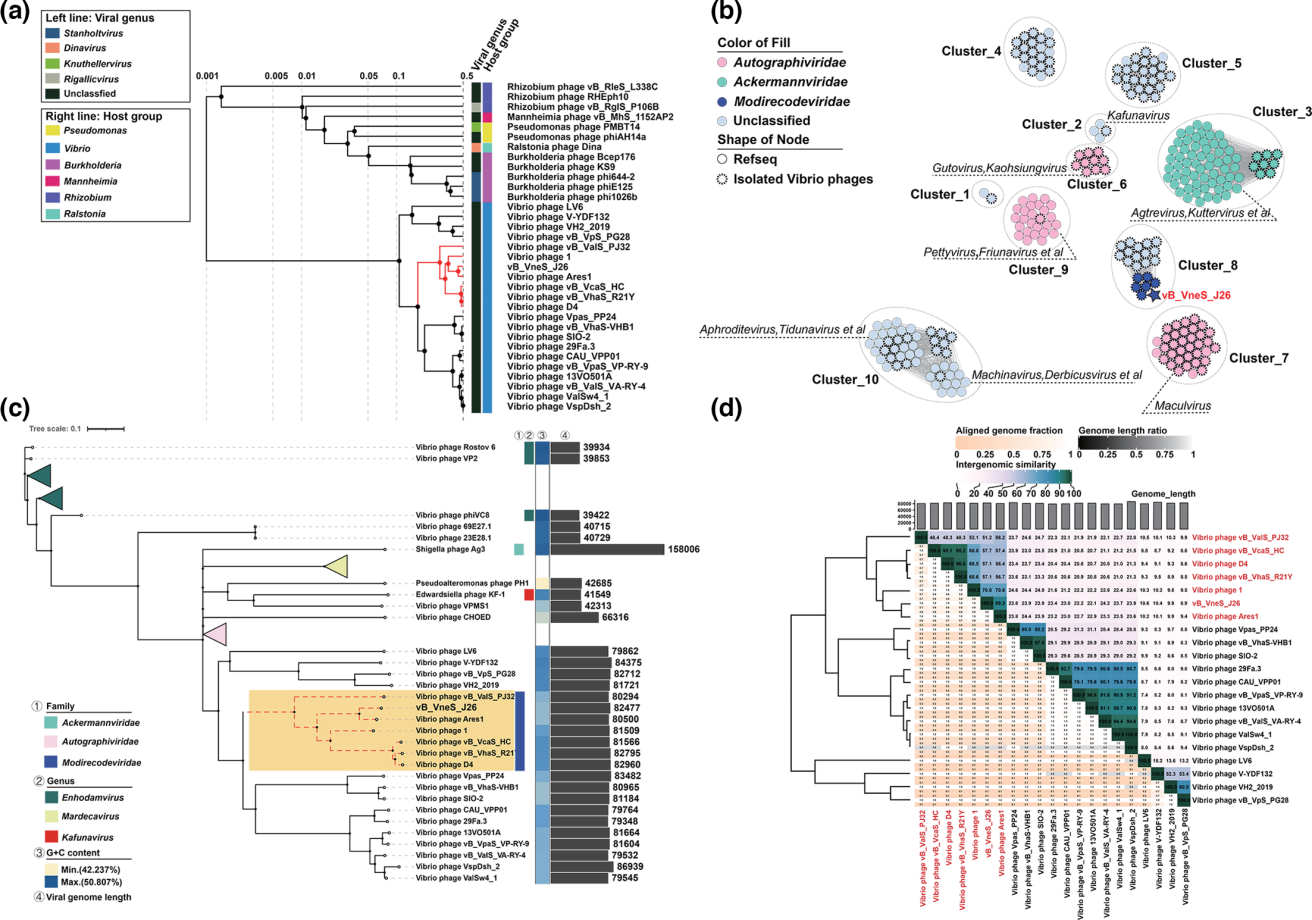
Phylogenetic and network analysis of vibriophage vB_VneS_J26 and related viruses. (**a**) The whole proteome tree was constructed using ViPTree based on tblastx to calculate whole-genome sequence similarities with related phages. *Modirecodeviridae* is highlighted with a solid red line. (**b**) A viral network based on gene content was generated between reference viral genome datasets from GenBank and vB_VneS_J26. Nodes represent viruses, dashed circles indicate viruses with *Vibrio* as their host and solid circles represent viruses with non-*Vibrio* hosts. The blue star denotes vB_VneS_J26. Viruses belonging to different families are represented in distinct colours. (**c**) Whole-genome phylogenetic tree was constructed for vB_VneS_J26 and related viruses from the viral network, based on nucleotide sequence similarity calculated using VICTOR. *Modirecodeviridae* is marked by a red dashed line. (**d**) A heatmap of ANI values for vB_VneS_J26 and related viruses was generated using VIRIDIC.

To determine the taxonomic status of vibriophage vB_VneS_J26 within dsDNA viruses, we constructed a viral proteome tree based on the complete genomes of 5,632 dsDNA viruses with prokaryotic hosts from ViPTree. We selected 32 complete viruses from a branch closely related to vB_VneS_J26 in ViPTree and constructed a proteome-based tree for these viruses, suggesting that vB_VneS_J26 and 20 vibriophages, belonging to the class *Caudoviricetes*, may have evolved from a common ancestor (Fig. 5a). vB_VneS_J26 clustered with six vibriophages (*Vibrio* phage vB_ValS_PJ32 (MT735629), *Vibrio* phage vB_VcaS_HC (MK559459), *Vibrio* phage D4 (OK381870), *Vibrio* phage vB_VhaS_R21Y (OR147960), *Vibrio* phage 1 (JF713456) and *Vibrio* phage Ares1 (MG720309), suggesting that vB_VneS_J26 and these six vibriophages represent a novel VC with unidirectional coding sequences, proposed family *Modirecodeviridae*. However, the evolutionary relationship between *Modirecodeviridae* and the remaining 14 vibriophages was unclear. To clarify this, we performed clustering analysis using vConTACT v2.0 (-pc-inflation 1.2, -link-prop 0.3 and -blast-evalue 1e-5) (Fig. 5b). A network map based on genomic content showed that *Modirecodeviridae*, along with these 14 vibriophages, belonged to cluster 8. Cluster 8 was not directly associated with any known viral families within *Caudoviricetes*, such as *Autographiviridae* and *Ackermannviridae*, confirming that these viruses do not belong to *Modirecodeviridae*. We then constructed a GBDP tree based on the complete viral nucleotide profiles of all members in the network (Fig. 5c). The genomes of cluster 8 ranged from 79,000 to 87,000 bp. *Modirecodeviridae* formed a distinct monophyletic branch, with seven vibriophages having genetic distances to their LCA of 0.246, 0.238, 0.225, 0.217, 0.292, 0.296 and 0.297. The remaining 14 vibriophages formed a separate monophyletic branch, indicating that they diverged from a common ancestor shared with *Modirecodeviridae*. We constructed a phylogenetic tree of the terminase LSUs from *Modirecodeviridae* and related viruses by IQ-TREE 2, showing that vB_VneS_J26 and six vibriophages formed a monophyletic group (Fig. S7). Additionally, we performed ANI analysis using VIRIDIC to define the genus and the species boundaries within *Modirecodeviridae* (Fig. 5d). Across *Modirecodeviridae*, pairwise ANI values ranged from 48.3% to 96%. Specifically, vB_VneS_J26 exhibited a maximum ANI of 89.3% with *Vibrio* phage Ares1 and a minimum of 56.2% with *Vibrio* phage vB_ValS_PJ32. In contrast, the ANI between *Modirecodeviridae* and the remaining 14 vibriophages was below 30%, insufficient to classify them within the same viral family. These results suggest that vB_VneS_J26 and the six vibriophages exhibit high genomic similarity and form a monophyletic group, indicating that they belong to a novel viral family cluster, *Modirecodeviridae*. Notably, *Vibrio* phage Ares1 and *Vibrio* phage 1, though in the same viral family and sharing significant genomic similarity with vB_VneS_J26, have distinct host specificities and interaction mechanisms. *Vibrio* phage Ares1 specifically infects *V. alginolyticus*, inducing host resistance by reducing LamB receptor protein expression and triggering metabolic reprogramming. Resistant host strains adjust their metabolism by enhancing the TCA cycle and amino acid metabolism to maintain energy homeostasis, forming a metabolic barrier against viral infection [18]. In contrast, *Vibrio* phage 1 infects *Vibrio harveyi*, exhibiting a unique unstable lysogeny-pseudolysogeny dynamic. Its genome exists as linear or loose multimers, prompting true lysogenic clones (TLCs) to spontaneously release phages in the late phase. However, this lysogenic state is unstable, with over 50% of TLCs losing viral DNA and converting to pseudolysogens during subculturing, yet retaining some lysogenic traits [81]. These two vibriophages represent different host interactions: *Vibrio* phage Ares1 drives the metabolic adaptation of hosts, whilst *Vibrio* phage 1 modulates the pathogenicity and competitiveness of hosts through phenotypic plasticity, offering distinct models for phage-host interaction research.

**Fig. 6.**
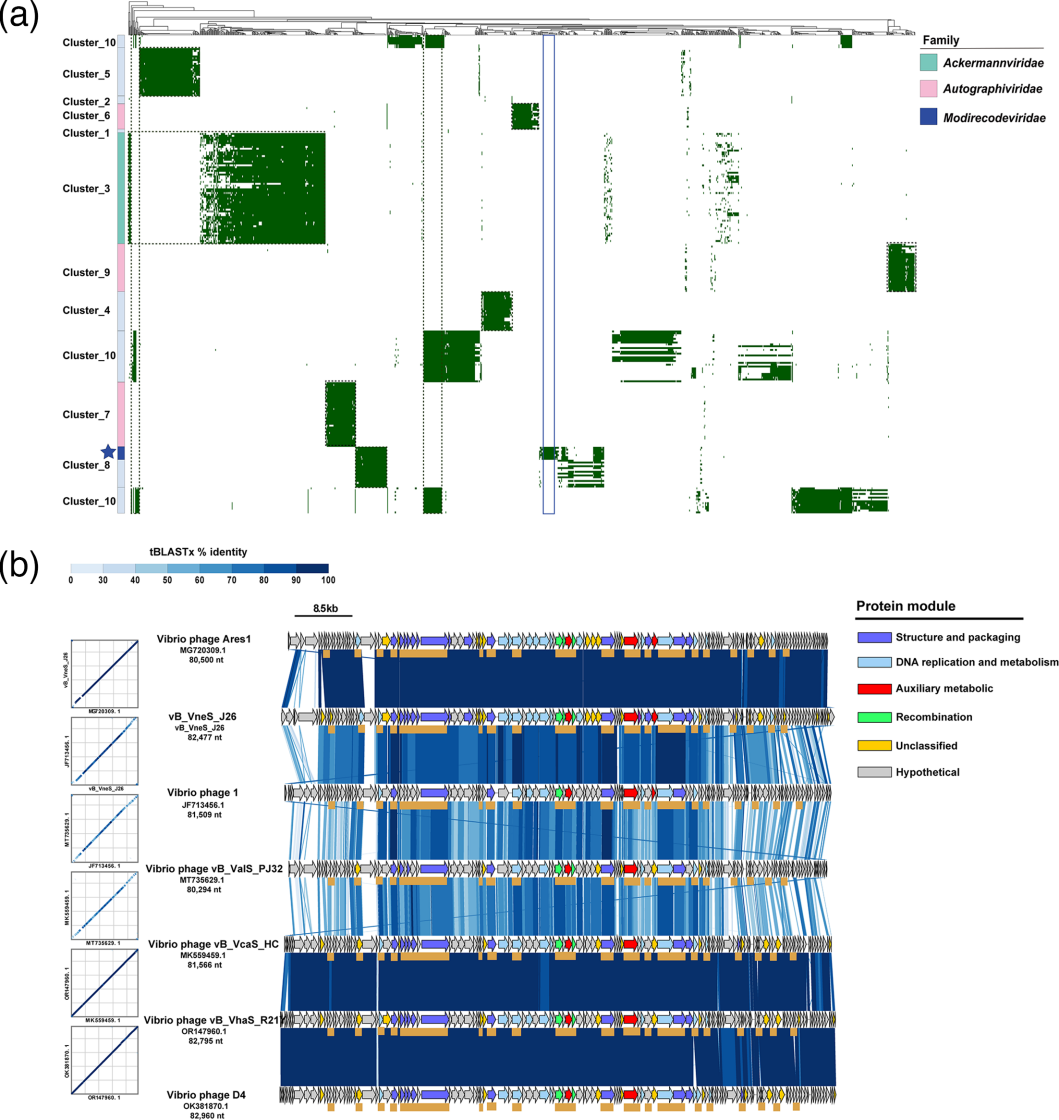
Comparative genomic analysis on vB_VneS_J26 to confirm core genes of *Modirecodeviridae* and gene covariation of members within this viral family. (**a**) Core gene clustering of *Modirecodeviridae* and related viruses. The core genes of viruses associated with *Modirecodeviridae* are analysed and presented in the heatmap. Protein clustering analysis was conducted using the MCL algorithm on *Modirecodeviridae* and reference viral genome datasets from GenBank. Different PCs represent various protein families. The solid blue lines indicate PCs unique to *Modirecodeviridae*, whilst the black dashed boxes represent core PCs shared amongst different VCs. The blue star marks *Modirecodeviridae*. (**b**) Gene covariance analysis of *Modirecodeviridae*. Different protein functional categories are shown in distinct colours, with the core genes of the viral family indicated by brown underlines.

A comparative genomic analysis was conducted to explore the core genes of candidate VCs and determine the taxonomic status of the viruses [82]. A viral dataset associated with *Modirecodeviridae* was selected for analysis, comprising all genomes that had weighted connections to vB_VneS_J26, as determined by the network map based on genomic content using vConTACT v2.0. Protein clustering was then performed on this dataset using the MCL. In Fig. 6(a), the core genes of different VCs are indicated by dashed lines, revealing distinct core gene modules across the VCs. In cluster 8, *Modirecodeviridae* and the other 14 vibriophages share several core PCs, including those involved in DNA replication and metabolism, structure and packaging, AMGs and recombination modules. The unique gene regions of *Modirecodeviridae* include proteins from three unclassified modules: TMhelix-containing protein, DUF1382 family protein and SEC-C motif protein. The remaining genes are hypothetical proteins, suggesting evolutionary independence for *Modirecodeviridae*, though most of these hypothetical genes require further investigation. Comparative genomic analysis results (Fig. 6b) demonstrate that the seven vibriophages within *Modirecodeviridae* exhibit unidirectional coding sequences and highly similar gene distribution modules, with five gene modules showing collinearity across the genomes. The core genes (underlined in brown in Fig. 6b) also display a similar distribution pattern throughout the genome, with continuous core genes in the structure and packaging, DNA replication and metabolism and recombination modules. *Modirecodeviridae* harbours identical AMG modules, including PPDK and rubredoxin-type fold protein, which help the host combat oxidative stress in the marine environment, promote carbon metabolism and generate energy, indicating coevolution with the host in similar environments. The core genes within the structural and packaging modules include tail-completion protein, tail proteins and tail-length tape measure protein, all of which are associated with the phage tail. These findings provide further evidence supporting the classification of *Modirecodeviridae* as viruses of siphovirus morphotype.

### Phylogeography of *Modirecodeviridae* in global ocean

**Fig. 7.**
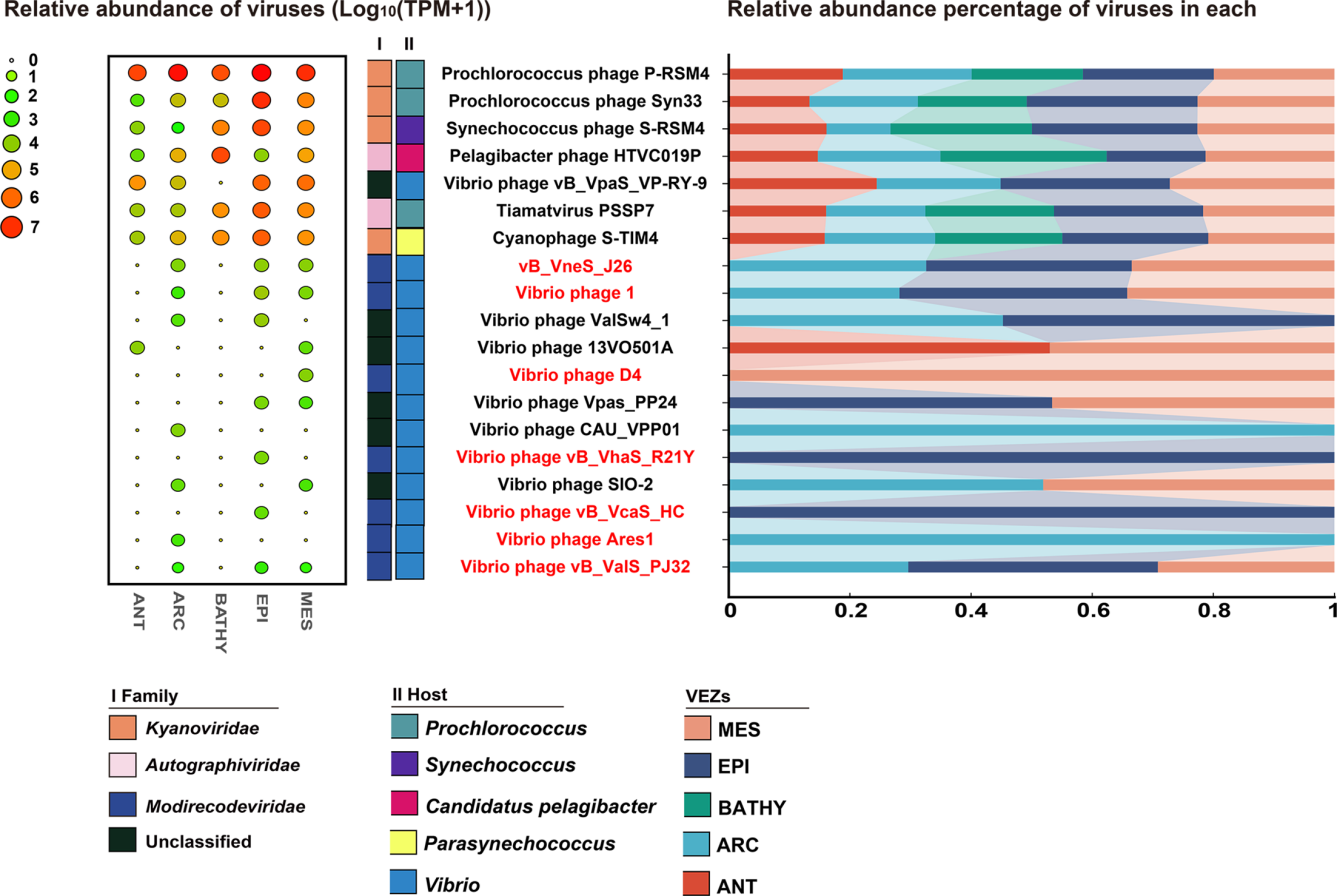
Relative abundances of *Modirecodeviridae*, reference viruses and other related vibriophages in the Global Ocean Viromes database (GOV2.0). Relative abundances of *Modirecodeviridae*, reference viruses and other related vibriophages were calculated amongst 154 viral metagenomes in the GOV 2.0 database. The values of relative abundances were normalized based on the database size for each VEZ and transformed using log_10_^(TPM+1)^. The bubble plot on the left displays the results after log_10_^(TPM+1)^ transformation, whilst the stacked bar chart on the right shows the percentage of each viral relative abundance within each marine VEZ.

To explore the phylogeography of *Modirecodeviridae* in the global ocean, we calculated the relative abundance of *Modirecodeviridae*, six related vibriophages, three *Synechococcus* phages, two *Prochlorococcus* phages, one *Pelagibacter* phage and one *Parasynechococcus* phage in the 154 viral metagenomes of the Global Ocean Viromes (GOV2.0) database. *Prochlorococcus* phage P-RSM4, *Prochlorococcus* phage Syn33, *Synechococcus* phage S-RSM4, *Pelagibacter* phage HTVC019P, Tiamatvirus PSSP7 and Cyanophage S-TIM4 exhibit high relative abundances across all five VEZs. None of the vibriophages showed relative abundance in the BATHY region. *Modirecodeviridae* were detected in the ARC, EPI and MES regions, with the relative abundance exceeding that of related vibriophages (except *Vibrio* phage vB_VpaS_VP-RY-9). Amongst *Modirecodeviridae*, vB_VneS_J26 had the highest relative abundance in the three VEZs. *Modirecodeviridae* exhibited varying distribution patterns: vB_VneS_J26, *Vibrio* phage 1 and *Vibrio* phage vB_ValS_PJ32 showed high relative abundances in three VEZs; *Vibrio* phage vB_VcaS_HC and *Vibrio* phage vB_VhaS_R21Y were present only in EPI; *Vibrio* phage D4 was detected in MES; and *Vibrio* phage Ares1 was found in ARC (Fig. 7). *Modirecodeviridae* are widely distributed in the ARC, and MES regions. This distribution pattern aligns with the occurrence of *Vibrio* in marine environments [83,84], suggesting that vibriophages may regulate the microbial abundance and community structure by infecting *Vibrio* abundances in surface seawaters, potentially impacting global biogeochemical cycles.

## Conclusions

*Vibrio* and vibriophages are vital members of microbial communities in the ocean, providing an appropriate model system for studying virus–host interactions. Vibriophages are widespread in aquatic environments, and *Vibrio* must endure continuous attacks from vibriophages to successfully propagate. vB_VneS_J26 is the first known phage to infect *V. neocaledonicus*. Genomic and phylogenetic analyses suggest its classification with six vibriophages into a novel viral family, proposed *Modirecodeviridae*. We have also identified the presence of PPDK in the genome, providing a structural basis for further research into the binding of PEP as the endogenous ligand and competitive inhibitor in PPDK catalytic reversible reactions. The isolation and characterization of phages within the novel viral family, proposed *Modirecodeviridae*, expand our current understanding of viral genomes, evolution and virus–host interactions in marine habitats.

## Supplementary material

10.1099/mgen.0.001403 Uncited Supplementary Material 1.

10.1099/mgen.0.001403 Uncited Supplementary Material 2.

## Abbreviations

AMGs: auxiliary metabolic genes
AMP: adenosine monophosphate
ANI: average nucleotide identity
ARC: arctic
BATHY: bathypelagic
CD: central domain
CsrA: carbon storage regulator A
dATP: deoxyadenosine triphosphate
dGTP: deoxyguanosine triphosphate
EMP: embden-meyerhof-parnas
EPI: epipelagic
GOV2: Global Ocean Virome
GTP: guanosine triphosphate
GTP: guanosine triphosphate
HJR: holliday junction resolvase
HJs: Holliday junctions
IMP: inosine monophosphate
iTOL: interactive Tree of Life
LCA: last common ancestor
LD: linker domain
MCL: Markov clustering algorithm
MES: mesopelagic
NBD: nucleotide ATP/AMP binding domain
NGS: next-generation sequencing
NMNAT: nicotinamide nucleotide adenylyltransferase
PBD: pyruvate binding domain
PCs: protein clusters
PDB: Protein Data Bank
PDC: pyruvate decarboxylase
PEP: phosphoenolpyruvate
PPDK: pyruvate phosphate dikinase
PTP: protein tyrosine phosphatase
RMSD: root mean square deviation
TCA: tricarboxylic acid
TEM: transmission electron microscopy
TIM: triose-phosphate isomerase
TLCs: true lysogenic clones
TPM: Transcripts Per Kilobase of exon model per Million mapped reads
VCs: viral clusters
VEZs: viral ecological zones
VICTOR: virus classification and tree building online resource
VIRIDIC: virus genome distance calculator

## References

[1] Mushegian A (2020). Are there 10^31^ virus particles on earth, or more, or fewer?. J Bacteriol.

[2] Breitbart M, Rohwer F (2005). Here a virus, there a virus, everywhere the same virus?. Trends Microbiol.

[3] Suttle CA (2007). Marine viruses—major players in the global ecosystem. Nat Rev Microbiol.

[4] Karl D, Letelier R, Tupas L, Dore J, Christian J (1997). The role of nitrogen fixation in biogeochemical cycling in the subtropical north pacific ocean. Nature.

[5] Rothman DH, Fournier GP, French KL, Alm EJ, Boyle EA (2014). Methanogenic burst in the end-permian carbon cycle. Proc Natl Acad Sci USA.

[6] Vincent AT, Derome N, Boyle B, Culley AI, Charette SJ (2017). Next-generation sequencing (NGS) in the microbiological world: how to make the most of your money. J Microbiol Methods.

[7] Sampaio A, Silva V, Poeta P, Aonofriesei F (2022). *Vibrio* spp.: life strategies, ecology, and risks in a changing environment. Diversity.

[8] Getz LJ (2022). Genome-wide investigation of *Vibrio parahaemolyticus* type III secretion system-1 regulation and chitin metabolism.

[9] Chbel A (2021). Marine biomolecules: a promising approach in therapy and biotechnology. Eur J Biological Research.

[10] Yu SX (2019). Isolation, identification, characterization, and sensitivity analysis of gut pathogenic *Vibrio* of *Urechis unicinctus*. Marine Sci.

[11] Geng Z (2022). The isolation and identification of a pathogenic *Vibrio neocaledonicus* from *Yesso scallop* (*Patinopecten yessoensis*). Invertebrate Surviv J.

[12] Chakraborty J CRC Press.

[13] Droubogiannis S, Katharios P (2025). Antimicrobial Resistance in Aquaculture and Aquatic Environments.

[14] Chastain CJ, Failing CJ, Manandhar L, Zimmerman MA, Lakner MM (2011). Functional evolution of C4 pyruvate, orthophosphate dikinase. J Exp Bot.

[15] Feng X, Yang C, Zheng W, Wen J (2014). Structural and evolutionary characteristics of pyruvate phosphate dikinase in Giardia lamblia and other amitochondriate protozoa. Chin Med J.

[16] Soto-Sánchez J, Pérez-Mora S, Ospina-Villa JD, Zavala-Ocampo LM (2024). Esters of quinoxaline-7-carboxylate 1, 4-di-N-oxide as potential inhibitors of glycolytic enzymes of *Entamoeba histolytica*: *in silico* approach. Curr Comput Aided Drug Des.

[17] Li C, Wang Z, Zhao J, Wang L, Xie G (2021). A novel vibriophage vb_vcas_hc containing lysogeny-related gene has strong lytic ability against pathogenic bacteria. Virol Sin.

[18] Skliros D, Kalatzis PG, Kalloniati C, Komaitis F, Papathanasiou S (2021). The development of bacteriophage resistance in *Vibrio alginolyticus* depends on a complex metabolic adaptation strategy. Viruses.

[19] Alfar HR, Pariser DN, Chanzu H, Joshi S, Coenen DM (2023). Protocol for optimizing production and quality control of infective EcoHIV virions. STAR Protoc.

[20] Roingeard P, Raynal P-I, Eymieux S, Blanchard E (2019). Virus detection by transmission electron microscopy: still useful for diagnosis and a plus for biosafety. Rev Med Virol.

[21] Wang H, Ren L, Liang Y, Zheng K, Guo R (2023). *Psychrobacter* phage encoding an antibiotics resistance gene represents a novel Caudoviral family. Microbiol Spectr.

[22] Frank JA, Reich CI, Sharma S, Weisbaum JS, Wilson BA (2008). Critical evaluation of two primers commonly used for amplification of bacterial 16S rRNA genes. Appl Environ Microbiol.

[23] Edgar RC (2022). Muscle5: high-accuracy alignment ensembles enable unbiased assessments of sequence homology and phylogeny. Nat Commun.

[24] Capella-Gutiérrez S, Silla-Martínez JM, Gabaldón T (2009). trimAl: a tool for automated alignment trimming in large-scale phylogenetic analyses. Bioinformatics.

[25] Piñeiro C, Pichel JC (2022). BigSeqKit: a parallel Big Data toolkit to process FASTA and FASTQ files at scale. Gigascience.

[26] Minh BQ, Schmidt HA, Chernomor O, Schrempf D, Woodhams MD (2020). IQ-TREE 2: new models and efficient methods for phylogenetic inference in the genomic era. Mol Biol Evol.

[27] Letunic I, Bork P (2024). Interactive Tree of Life (iTOL) v6: recent updates to the phylogenetic tree display and annotation tool. Nucleic Acids Res.

[28] Li JK, Sun C, Li S, Cui YS, Wei YL (2015). AASRI International Conference on Industrial Electronics and Applications (IEA 2015).

[29] Scientific, T (2010).

[30] Bolger AM, Lohse M, Usadel B (2014). Trimmomatic: a flexible trimmer for illumina sequence data. Bioinformatics.

[31] Xu M, Guo L, Gu S, Wang O, Zhang R (2020). TGS-GapCloser: a fast and accurate gap closer for large genomes with low coverage of error-prone long reads. Gigascience.

[32] Nayfach S, Camargo AP, Schulz F, Eloe-Fadrosh E, Roux S (2021). CheckV assesses the quality and completeness of metagenome-assembled viral genomes. Nat Biotechnol.

[33] Garneau JR, Depardieu F, Fortier L-C, Bikard D, Monot M (2017). PhageTerm: a tool for fast and accurate determination of phage termini and packaging mechanism using next-generation sequencing data. Sci Rep.

[34] Aziz RK, Bartels D, Best AA, DeJongh M, Disz T (2008). The RAST server: rapid annotations using subsystems technology. BMC genom.

[35] Hyatt D, Chen G-L, Locascio PF, Land ML, Larimer FW (2010). Prodigal: prokaryotic gene recognition and translation initiation site identification. BMC Bioinf.

[36] Madeira F, Pearce M, Tivey ARN, Basutkar P, Lee J (2022). Search and sequence analysis tools services from EMBL-EBI in 2022. Nucleic Acids Res.

[37] Kanehisa M, Sato Y, Morishima K (2016). BlastKOALA and ghostkoala: KEGG tools for functional characterization of genome and metagenome sequences. J Mol Biol.

[38] Gabler F, Nam S-Z, Till S, Mirdita M, Steinegger M (2020). Protein sequence analysis using the MPI bioinformatics toolkit. Curr Protoc Bioinf.

[39] Chan PP, Lowe TM (2019).

[40] Lu J, Salzberg SL (2020). SkewIT: the skew index test for large-scale GC skew analysis of bacterial genomes. PLoS Comput Biol.

[41] Teeling H, Meyerdierks A, Bauer M, Amann R, Glöckner FO (2004). Application of tetranucleotide frequencies for the assignment of genomic fragments. Environ Microbiol.

[42] Duhaime MB, Wichels A, Waldmann J, Teeling H, Glöckner FO (2011). Ecogenomics and genome landscapes of marine *Pseudoalteromonas* phage H105/1. ISME J.

[43] Abramson J, Adler J, Dunger J, Evans R, Green T (2024). Accurate structure prediction of biomolecular interactions with AlphaFold 3. Nature.

[44] Holm L, Laakso LM (2016). Dali server update. Nucleic Acids Res.

[45] Yuan S, Chan HCS, Hu Z (2017). Using PyMOL as a platform for computational drug design. WIREs Comput Mol Sci.

[46] John B (2003). Comparative protein structure modeling by iterative alignment, model building and model assessment. Nucleic Acids Res.

[47] Mirdita M, Steinegger M, Breitwieser F, Söding J, Levy Karin E (2021). Fast and sensitive taxonomic assignment to metagenomic contigs. Bioinformatics.

[48] Bin Jang H, Bolduc B, Zablocki O, Kuhn JH, Roux S (2019). Taxonomic assignment of uncultivated prokaryotic virus genomes is enabled by gene-sharing networks. Nat Biotechnol.

[49] Nepusz T, Yu H, Paccanaro A (2012). Detecting overlapping protein complexes in protein-protein interaction networks. Nat Methods.

[50] Nishimura Y, Yoshida T, Kuronishi M, Uehara H, Ogata H (2017). ViPTree: the viral proteomic tree server. Bioinformatics.

[51] Meier-Kolthoff JP, Göker M (2017). VICTOR: genome-based phylogeny and classification of prokaryotic viruses. Bioinformatics.

[52] Moraru C, Varsani A, Kropinski AM (2020). VIRIDIC-a novel tool to calculate the intergenomic similarities of prokaryote-infecting viruses. Viruses.

[53] Li H (2018). Minimap2: pairwise alignment for nucleotide sequences. Bioinformatics.

[54] Sunagawa S, Acinas SG, Bork P, Bowler C, Acinas SG (2020). Tara Oceans: towards global ocean ecosystems biology. Nat Rev Microbiol.

[55] Gregory AC, Zayed AA, Conceição-Neto N, Temperton B, Bolduc B (2019). Marine DNA viral macro-and microdiversity from pole to pole. Cell.

[56] Reygondeau G, Guidi L, Beaugrand G, Henson SA, Koubbi P (2018). Global biogeochemical provinces of the mesopelagic zone. J Biogeogr.

[57] Zhao Y, Temperton B, Thrash JC, Schwalbach MS, Vergin KL (2013). Abundant SAR11 viruses in the ocean. Nature.

[58] Kang I, Oh H-M, Kang D, Cho J-C (2013). Genome of a SAR116 bacteriophage shows the prevalence of this phage type in the oceans. Proc Natl Acad Sci USA.

[59] Oliveira L, Tavares P, Alonso JC (2013). Headful DNA packaging: bacteriophage SPP1 as a model system. Virus Res.

[60] Sivapragasam S, Grove A (2019). The link between purine metabolism and production of antibiotics in *Streptomyces*. Antibiotics.

[61] Cheng H, Shen N, Pei J, Grishin NV (2004). Double-stranded DNA bacteriophage prohead protease is homologous to herpesvirus protease. Protein Sci.

[62] Sanjuán R, Pereira-Gómez M, Risso J (2016). Genome Stability.

[63] Pérez-Losada M, Arenas M, Galán JC, Palero F, González-Candelas F (2015). Recombination in viruses: mechanisms, methods of study, and evolutionary consequences. Infect Genet Evol.

[64] Campa AR, Smith LM, Hampton HG, Sharma S, Jackson SA (2021). The Rsm (Csr) post-transcriptional regulatory pathway coordinately controls multiple CRISPR-Cas immune systems. Nucleic Acids Res.

[65] Potts AH, Guo Y, Ahmer BMM, Romeo T (2019). Role of CsrA in stress responses and metabolism important for *Salmonella* virulence revealed by integrated transcriptomics. PLoS One.

[66] Ibrahim AMA, Choi JY, Je YH, Kim Y (2007). Protein tyrosine phosphatases encoded in *Cotesia plutellae* bracovirus: sequence analysis, expression profile, and a possible biological role in host immunosuppression. Dev Comp Immunol.

[67] Isaev AB, Musharova OS, Severinov KV (2021). Microbial arsenal of antiviral defenses – Part I. Biochemistry.

[68] Murphy J, Mahony J, Ainsworth S, Nauta A, van Sinderen D (2013). Bacteriophage orphan DNA methyltransferases: insights from their bacterial origin, function, and occurrence. Appl Environ Microbiol.

[69] Miller ES, Heidelberg JF, Eisen JA, Nelson WC, Durkin AS (2003). Complete genome sequence of the broad-host-range vibriophage KVP40: comparative genomics of a T4-related bacteriophage. J Bacteriol.

[70] Lee JY, Li Z, Miller ES (2017). *Vibrio* phage KVP40 encodes a functional NAD^+^ salvage pathway. J Bacteriol.

[71] Skliros D, Kalatzis PG, Katharios P, Flemetakis E (2016). Comparative functional genomic analysis of two *Vibrio* phages reveals complex metabolic interactions with the host cell. Front Microbiol.

[72] Ueda C (2022). The Biophysical and Spectroscopic Characterization of Redox-Active Metallocofactors in a Viral Fe-S Protein and a Diiron HD-Domain Phosphohydrolase.

[73] Zhao J, Jing H, Wang Z, Wang L, Jian H (2022). Novel viral communities potentially assisting in carbon, nitrogen, and sulfur metabolism in the upper slope sediments of Mariana Trench. mSystems.

[74] van Zyl LJ, Schubert W-D, Tuffin MI, Cowan DA (2014). Structure and functional characterization of pyruvate decarboxylase from *Gluconacetobacter diazotrophicus*. BMC Struct Biol.

[75] Lin W, Liu Y, Molho M, Zhang S, Wang L (2019). Co-opting the fermentation pathway for tombusvirus replication: compartmentalization of cellular metabolic pathways for rapid ATP generation. PLoS Pathog.

[76] Nagy PD, Lin W (2020). Taking over cellular energy-metabolism for TBSV replication: the high ATP requirement of an RNA virus within the viral replication organelle. Viruses.

[77] Minges A, Ciupka D, Winkler C, Höppner A, Gohlke H (2017). Structural intermediates and directionality of the swiveling motion of pyruvate phosphate dikinase. Sci Rep.

[78] Ciupka D, Gohlke H (2017). On the potential alternate binding change mechanism in a dimeric structure of pyruvate phosphate dikinase. Sci Rep.

[79] Herzberg O, Chen CCH, Liu S, Tempczyk A, Howard A (2002). Pyruvate site of pyruvate phosphate dikinase: crystal structure of the enzyme− phosphonopyruvate complex, and mutant analysis. Biochemistry.

[5] Stephen P, Vijayan R, Bhat A, Subbarao N, Bamezai RNK (2008). Molecular modeling on pyruvate phosphate dikinase of *Entamoeba histolytica* and *in silico* virtual screening for novel inhibitors. J Comput Aided Mol Des.

[81] Khemayan K, Pasharawipas T, Puiprom O, Sriurairatana S, Suthienkul O (2006). Unstable lysogeny and pseudolysogeny in *Vibrio* harveyi siphovirus-like phage 1. Appl Environ Microbiol.

[82] Chan JZ-M, Millard AD, Mann NH, Schäfer H (2014). Comparative genomics defines the core genome of the growing N4-like phage genus and identifies N4-like Roseophage specific genes. Front Microbiol.

[83] Thompson FL, Iida T, Swings J (2004). Biodiversity of *Vibrios*. Microbiol Mol Biol Rev.

[84] Jøstensen J (1997). High prevalence of polyunsaturated-fatty-acid producing bacteria in arctic invertebrates. FEMS Microbiol Lett.

