# Supplementary material for: Isolation, complete characterization and phylogeography of the first bacteriophage against Vibrio neocaledonicus, which encodes a pyruvate phosphate dikinase and represents a novel viral family
Source: Microb Genom. 2025 Apr 28;11(4):001403. doi: 10.1099/mgen.0.001403 (PMC12038007; doi:10.1099/mgen.0.001403)
Supplement: Uncited Supplementary Material 1. [file mgen-11-01403-s001.pdf]

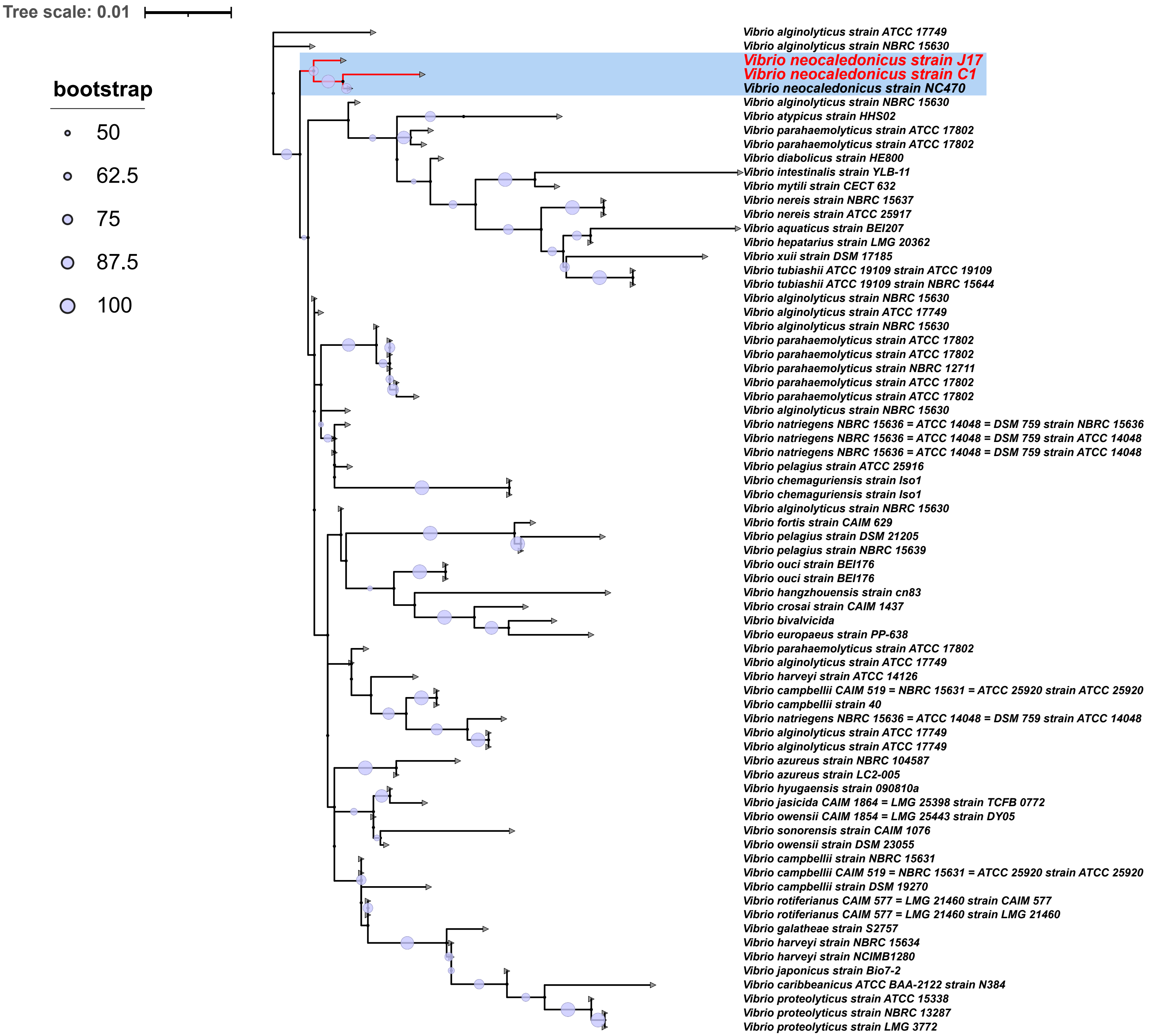

Fig S1. Phylogenetic tree based on the 16S rRNA gene sequences of C1, J17, and 72 other *Vibrio* reference strains.

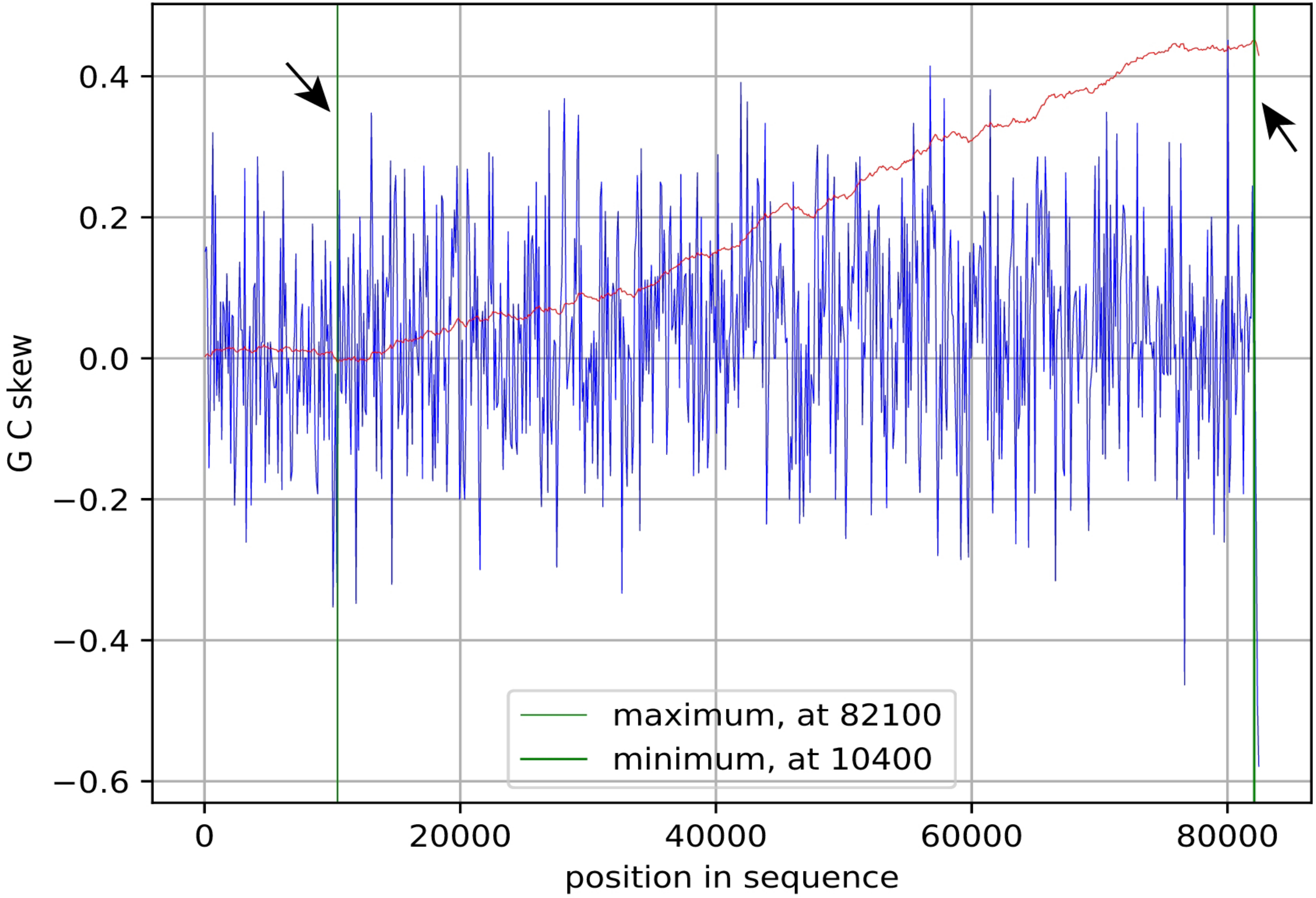

Fig S2. Cumulated GC-skew analysis of vB_VneS_J26. The GC-skew of the genome sequence was analyzed using a 100 bp window size and step size to calculate the global minimum and maximum values. The GC-skew and cumulated GC-skew are shown as blue and red lines, respectively.

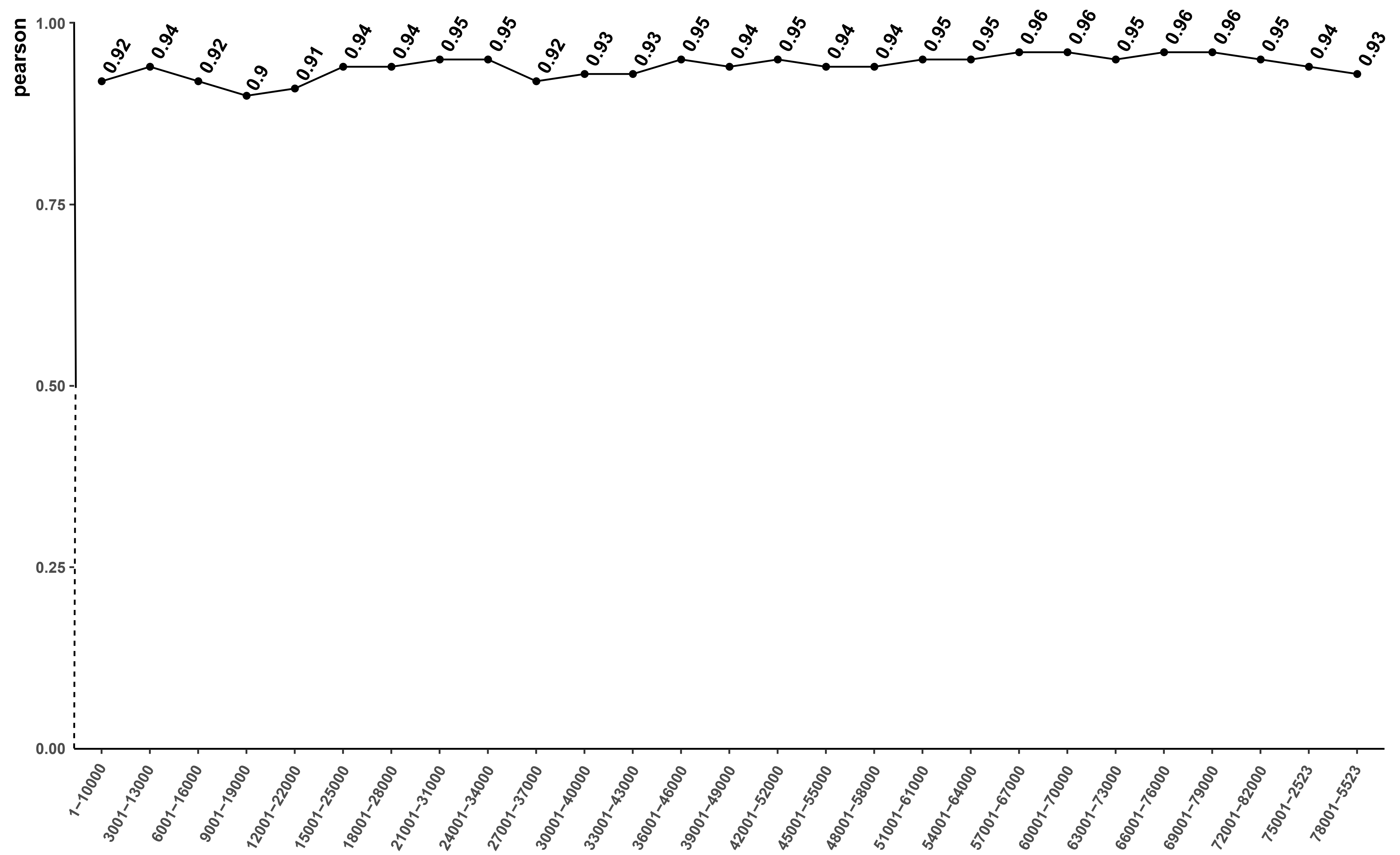

Fig S3. Tetra nucleotide correlation analysis of the vB_VneS_J26. The tetra nucleotide correlation of each 10 kbp genomic segment with the entire viral genome was displayed using a 10 kbp window size and a 3 kbp step size.

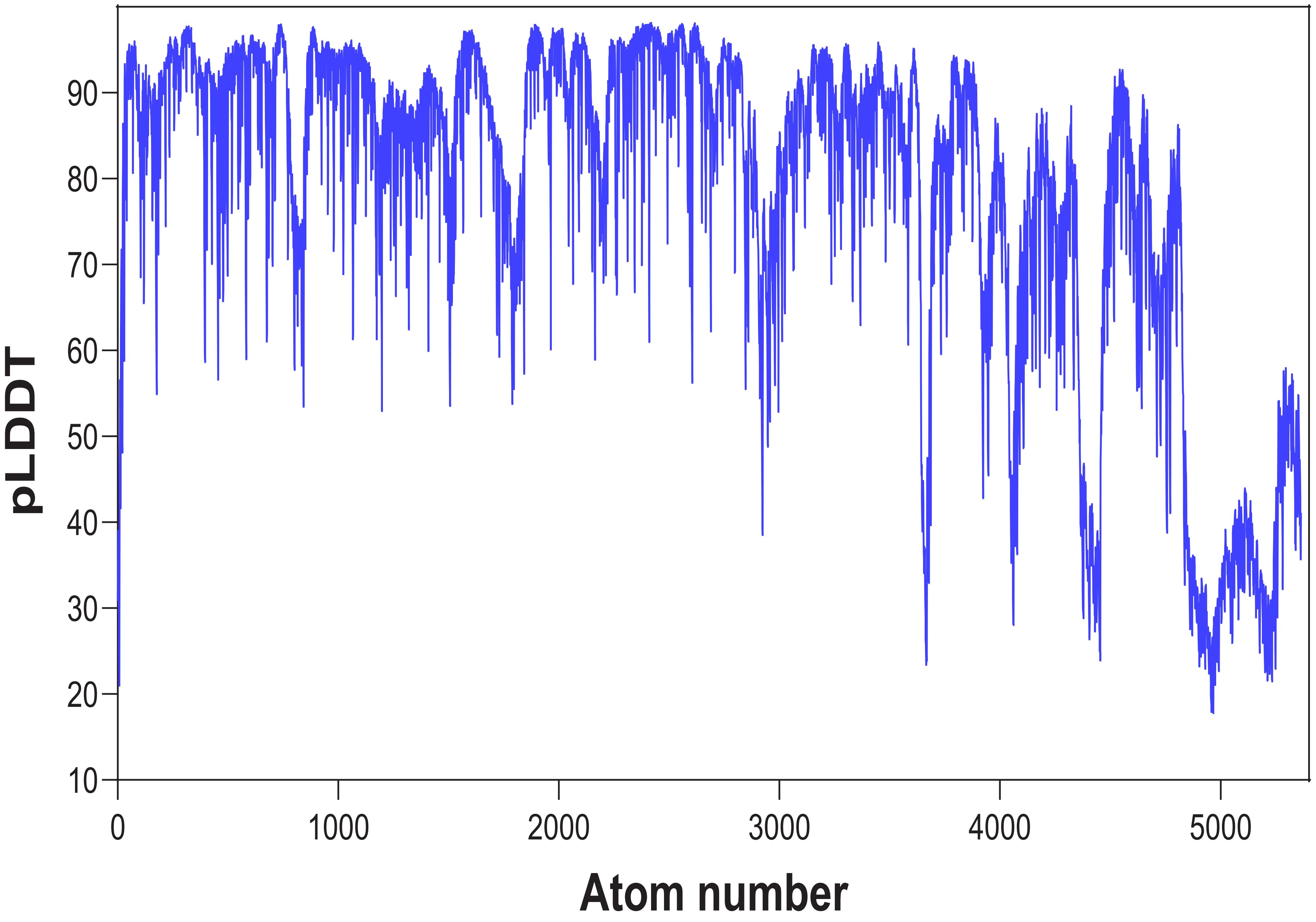

Fig S4. The per-atom Local-Distance Difference Test (pLDDT) of the ORF98 model.

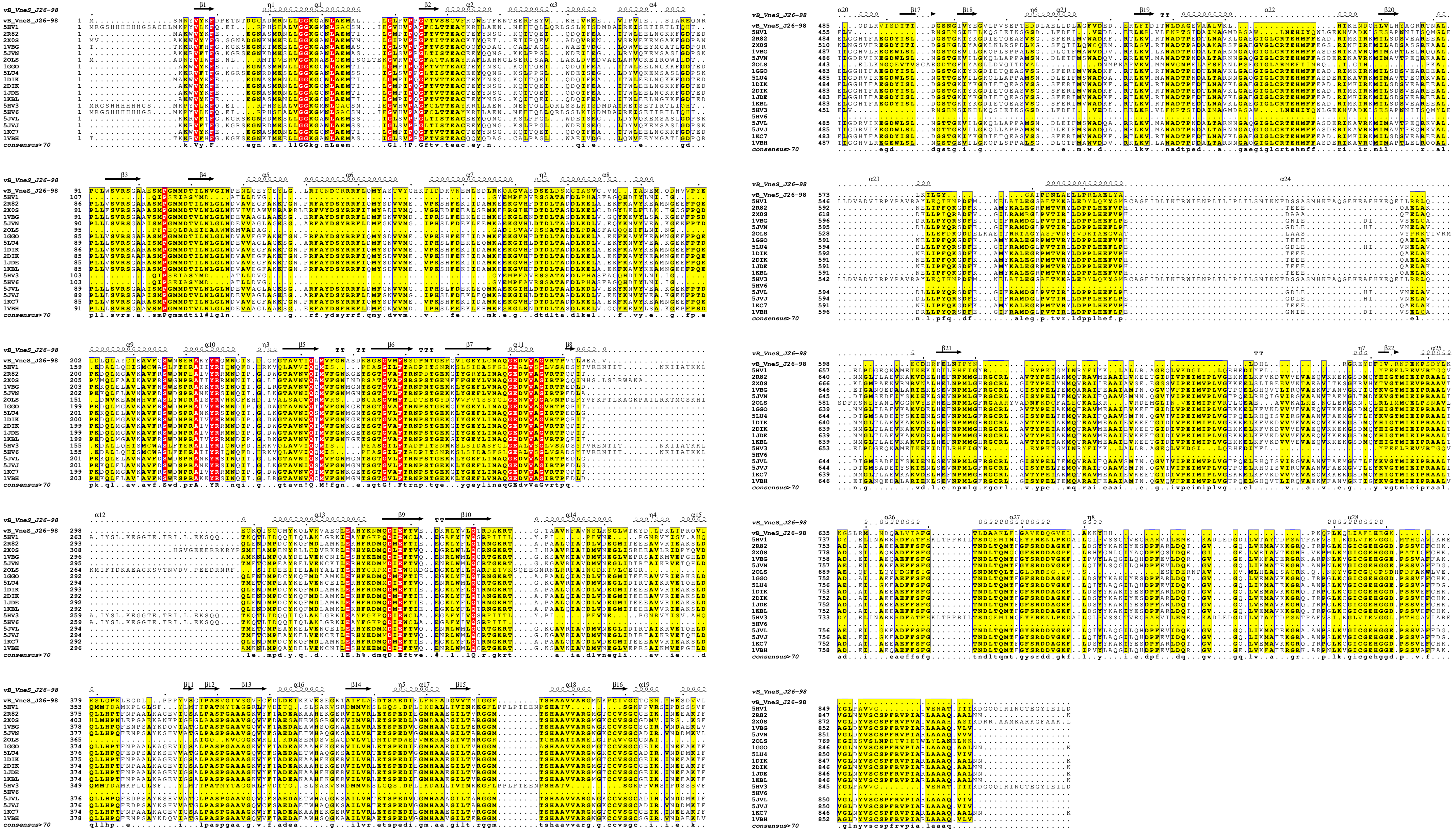

Fig. S5 The conserved positions of PPDK and ORF98 in primary structures are highlighted. The yellow regions indicate positions with over 70% similarity to ORF98, while the red regions denote positions identical to those in ORF98.

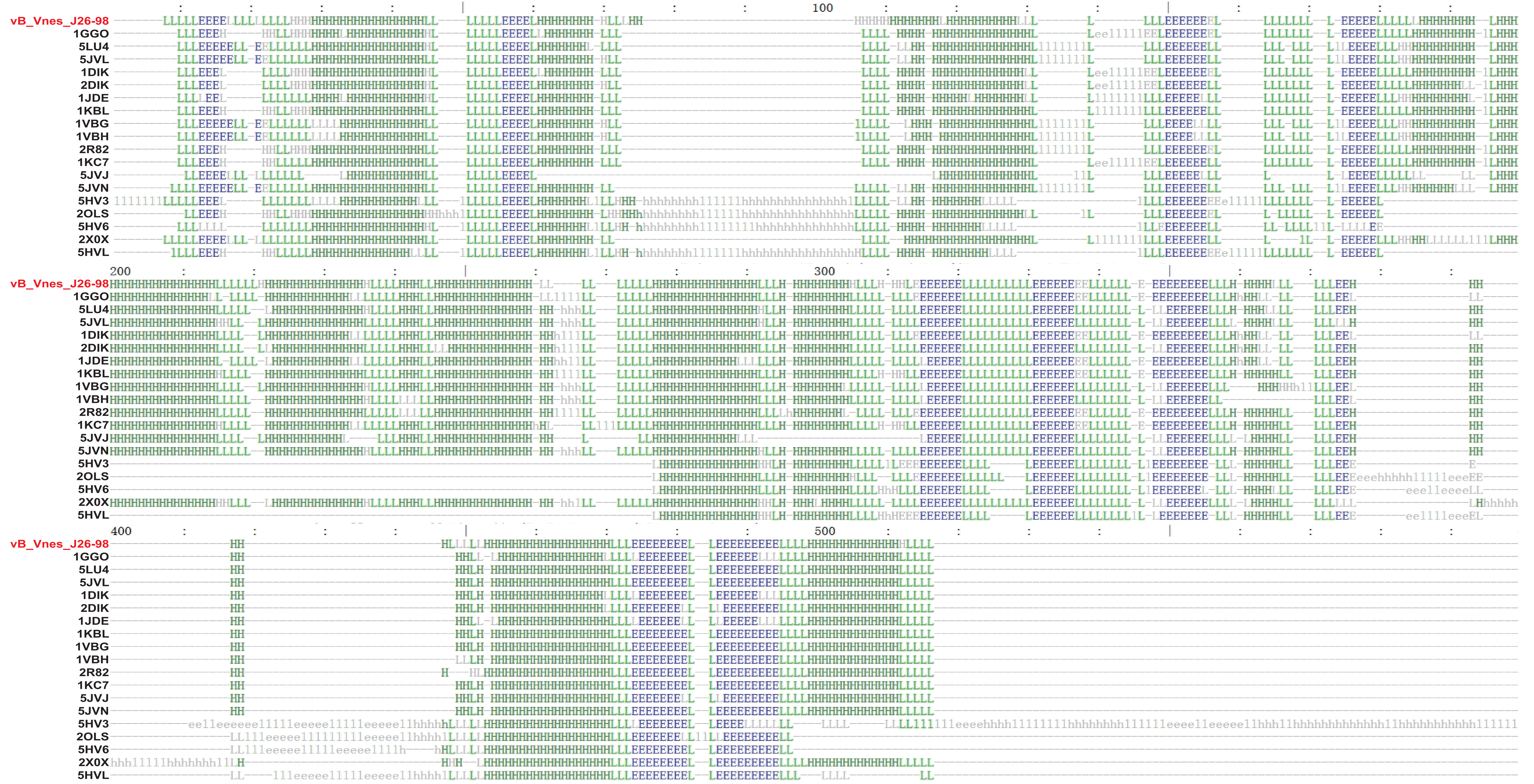

Fig S6. DSSP prediction of secondary structure assignments for ORF98 and 18 PPDKs (H/h: helix, E/e: strand, L/l: loop).

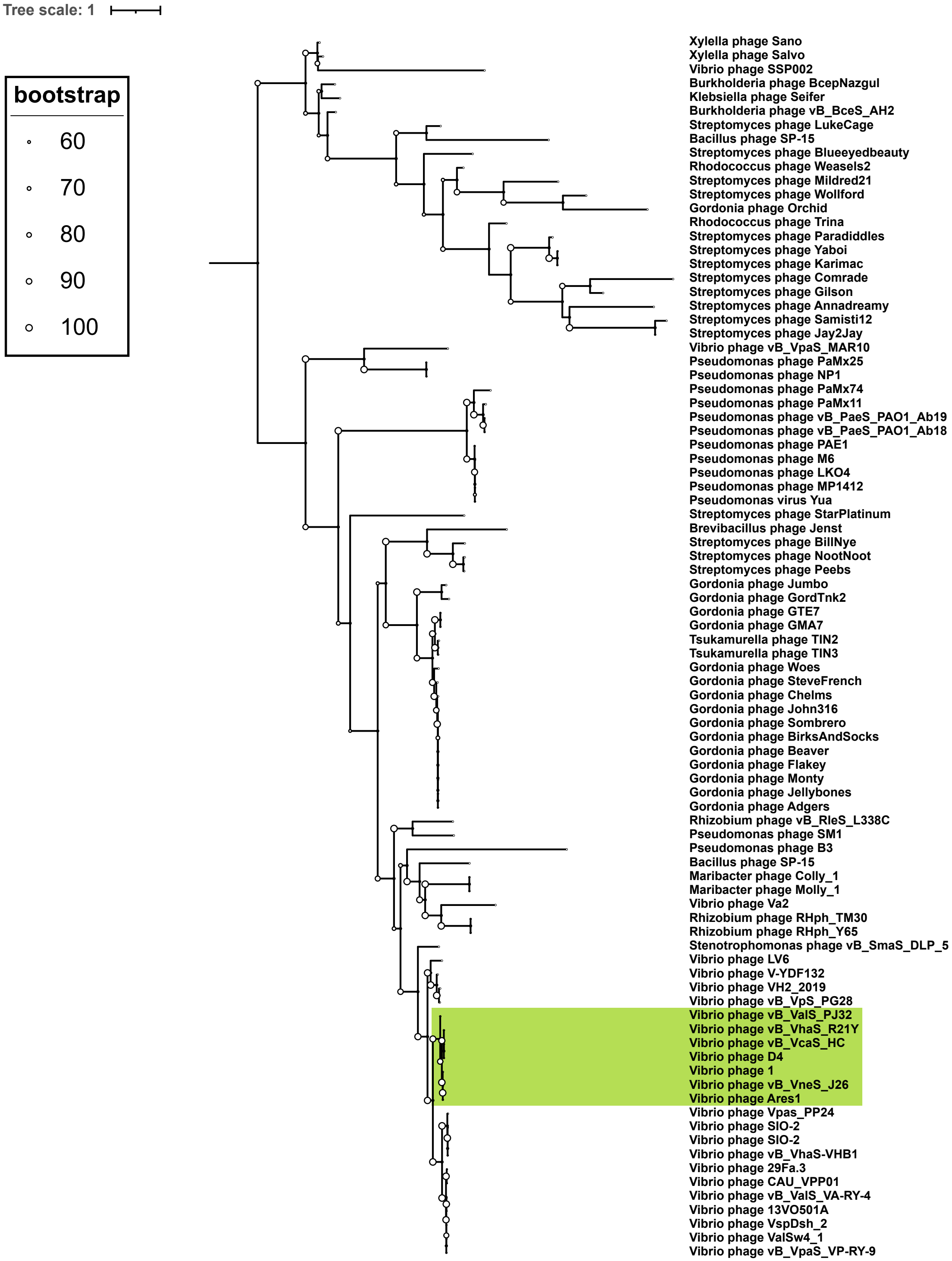

Fig S7. The phylogenic tree of vB_VneS_J26 is based on terminase large subunits.
